# mTOR inhibition amplifies the anti-lymphoma effect of PI3Kβ/δ blockage in diffuse large B-cell lymphoma

**DOI:** 10.1038/s41375-022-01749-0

**Published:** 2022-11-09

**Authors:** Wendan Xu, Philipp Berning, Tabea Erdmann, Michael Grau, Nardjas Bettazová, Myroslav Zapukhlyak, Fabian Frontzek, Corinna Kosnopfel, Peter Lenz, Michael Grondine, Brandon Willis, James T. Lynch, Pavel Klener, Stephan Hailfinger, Simon T. Barry, Georg Lenz

**Affiliations:** 1 Department of Hematology, Oncology and Pneumology, University Hospital Muenster, Muenster, Germany; 2 Institute of Pathological Physiology, First Faculty of Medicine, Charles University, Prague, Czech Republic; 3 Department of Medical Genetics, Third Faculty of Medicine, Charles University, Prague, Czech Republic; 4 Department of Physics, University of Marburg, Marburg, Germany; 5 LOEWE Center for Synthetic Microbiology, Marburg, Germany; 6 Bioscience, Early Oncology, AstraZeneca, Boston, MA USA; 7 Bioscience, Early Oncology, AstraZeneca, Cambridge, UK; 8 First Department of Internal Medicine - Department of Hematology, University General Hospital and First Faculty of Medicine, Charles University, Prague, Czech Republic

**Keywords:** B-cell lymphoma, Targeted therapies

## Abstract

Diffuse large B-cell lymphoma (DLBCL) is an aggressive disease that exhibits constitutive activation of phosphoinositide 3-kinase (PI3K) driven by chronic B-cell receptor signaling or PTEN deficiency. Since pan-PI3K inhibitors cause severe side effects, we investigated the anti-lymphoma efficacy of the specific PI3Kβ/δ inhibitor AZD8186. We identified a subset of DLBCL models within activated B-cell–like (ABC) and germinal center B-cell–like (GCB) DLBCL that were sensitive to AZD8186 treatment. On the molecular level, PI3Kβ/δ inhibition decreased the pro-survival NF-κB and AP-1 activity or led to downregulation of the oncogenic transcription factor MYC. In AZD8186-resistant models, we detected a feedback activation of the PI3K/AKT/mTOR pathway following PI3Kβ/δ inhibition, which limited AZD8186 efficacy. The combined treatment with AZD8186 and the mTOR inhibitor AZD2014 overcame resistance to PI3Kβ/δ inhibition and completely prevented outgrowth of lymphoma cells in vivo in cell line- and patient-derived xenograft mouse models. Collectively, our study reveals that subsets of DLBCLs are addicted to PI3Kβ/δ signaling and thus identifies a previously unappreciated role of the PI3Kβ isoform in DLBCL survival. Furthermore, our data demonstrate that combined targeting of PI3Kβ/δ and mTOR is effective in all major DLBCL subtypes supporting the evaluation of this strategy in a clinical trial setting.

## Introduction

Diffuse large B-cell lymphoma (DLBCL) represents the most common malignant lymphoma subtype in adults accounting for approximately 30% of all lymphoma cases [1]. DLBCL is a heterogeneous diagnostic category with respect to biology, genetic aberrations, and clinical presentation [2]. Although in more than two-thirds of patients a sustainable response is achieved by first-line treatment with rituximab in combination with cyclophosphamide, doxorubicin, vincristine, and prednisolone (R-CHOP), the remaining patients who either do not respond to R-CHOP or relapse after an initial response suffer from dismal prognosis, highlighting the need for novel therapeutic approaches [3–7].

Gene expression profiling (GEP) identified two major molecular subtypes of DLBCL in adults: activated B-cell–like (ABC) and germinal center B-cell–like (GCB) DLBCL [8]. In particular, ABC DLBCL patients are characterized by inferior prognosis compared to the GCB DLBCL patients [9]. Aberrant activation of the phosphoinositide 3-kinase (PI3K)/AKT/mTOR pathway is observed in a significant subset of DLBCL samples and is driven by chronic activated B-cell receptor (BCR) signaling or the loss of phosphatase and tensin homolog (PTEN) expression [10–13]. Whereas chronic BCR signaling, e.g. caused by mutations in *CD79B*, is a hallmark of ABC DLBCL, PTEN deficiency is frequently observed in the GCB subtype [13, 14].

Class I PI3Ks catalyze the conversion of phosphatidylinositol (4,5)-bisphosphate (PIP_2_) into phosphatidylinositol (3,4,5)-trisphosphate (PIP_3_), which serves as second messenger and contributes to the recruitment and subsequent activation of proteins containing a pleckstrin homology domain, such as AKT and BTK [15]. All class I PI3Ks are heterodimers comprising a regulatory and a catalytic subunit [16]. Four separate genes encode the catalytic subunits that are either ubiquitously expressed (α and β isoform) or predominantly in leukocytes only (γ and δ isoform) [16].

As pan-PI3K inhibitors cause severe side effects, isoform-specific inhibitors have been evaluated in various clinical trials in different hematologic and solid malignancies [17–21]. In patients with relapsed/refractory DLBCL the PI3Kδ inhibitor idelalisib showed only modest activity suggesting that more than one PI3K isoform has to be inhibited either due to functional redundancies or to prevent feedback mechanisms [22]. Accordingly, several preclinical and early clinical studies have demonstrated that combined inhibition of the PI3Kα/δ isoforms shows promising results, especially in the treatment of ABC DLBCL patients [12, 19, 23, 24]. In contrast, the role of the PI3Kβ isoform in lymphoma survival and proliferation is currently poorly understood. Thus, we sought to elucidate in this study the importance of PI3Kβ/δ signaling in DLBCL models in vitro and in vivo as well as to unravel the molecular mechanisms underlying the efficacy of AZD8186, a potent inhibitor of PI3Kβ with activity against PI3Kδ signaling [25]. We further pinpoint potential synergistic pharmacological combinations that sensitize primarily resistant DLBCL models towards PI3Kβ/δ inhibition.

## Materials and methods

### Cell culture, retroviral constructs, and transductions

Protocols are available in the Supplementary Information.

### In vitro viability assay

For viability assays, DLBCL cell lines were incubated with DMSO or different concentrations of indicated inhibitors in a total volume of 100 µL in 96-well plates. After incubation for 120 h, cell viability was measured using the CellTiter-Glo® Luminescent Cell Viability Assay (Promega, Madison, WI, USA) according to the manufacturer’s instructions. Luminescence was measured on a Victor Multimode Reader (PerkinElmer, Waltham, MA, USA). All experiments were reproduced at least two times for the indicated cell lines.

### Gene expression profiling and quantitative PCR

Protocols are available in the Supplementary Information.

### ELISA

Secreted interleukin-6 (IL6) and IL10 levels were quantified using human IL6 and IL10 Quantikine® ELISA immunoassays (R&D Systems, Minneapolis, MN, USA) according to manufacturer’s instructions.

### Western blotting

Protocols are available in the Supplementary Information.

### Cell cycle, apoptosis, and proliferation analysis

Cell cycle analysis of fixed cells was performed on a cell analyzer NucleoCounter NC-250 (ChemoMetec, Allerod, Denmark) using the Two-step cell cycle analysis kit according to the manufacturer’s application note. Respective experiments were repeated at least three times.

To assess cell proliferation, DLBCL cell lines were stained with Carboxyfluorescein succinimidyl ester (CFSE, eBioscience, San Diego, CA, USA) prior to treatment with DMSO or indicated inhibitors. CFSE dilutions were measured on a flow cytometer after five days of incubation with DMSO or inhibitors. The mean fluorescence intensity of the inhibitor treated group was normalized to the DMSO group. Apoptosis analysis was performed in DLBCL cell lines 48 h after incubation with DMSO or indicated inhibitors using the FITC Annexin V Apoptosis Detection Kit I (Beckton Dickinson, Franklin Lakes, NJ, USA) according to the manufacturer’s recommendations. Annexin V-stained cells were measured by flow cytometry. The fractions of apoptotic cells of each treatment group were normalized to the DMSO group.

### In vivo xenograft mouse studies

Protocols are available in the Supplementary Information.

### Synergy analyses

Protocols are available in the Supplementary Information.

## Results

### PI3Kβ/δ inhibition affects the growth of selected DLBCL cell lines

**Fig. 1:**
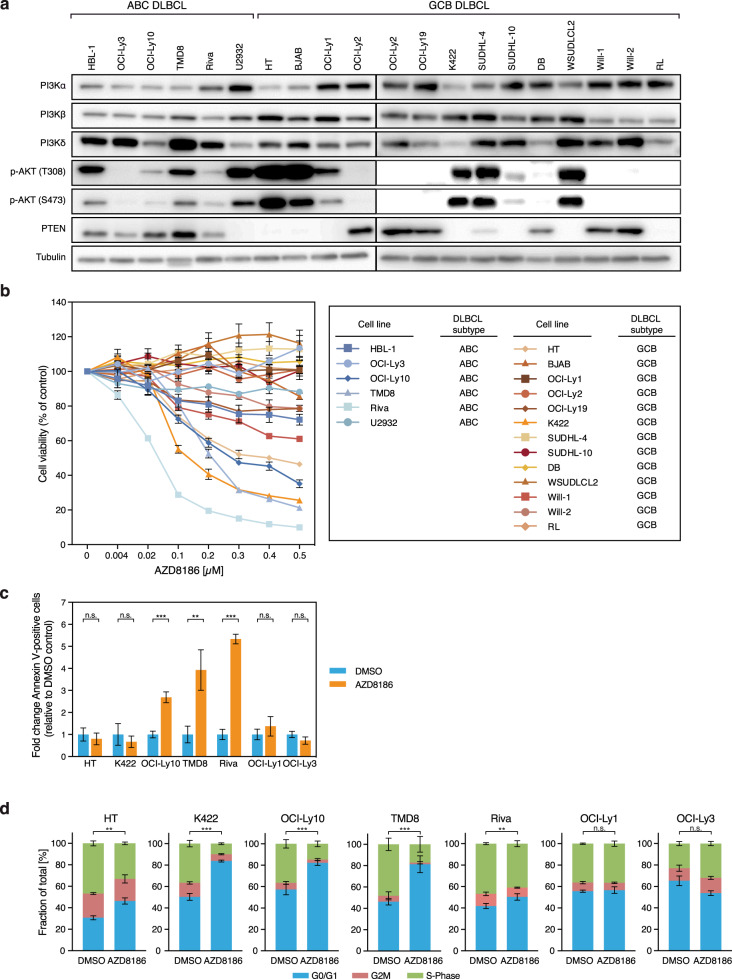
Expression of PI3K isoforms and efficacy of the PI3Kβ/δ inhibitor AZD8186 in DLBCL subtypes. **a** Expression of PI3Kα/β/δ and PTEN, as well as AKT phosphorylation was visualized by immunoblotting in the indicated six ABC and 13 GCB DLBCL cell lines. Tubulin served as loading control. **b** Cell viability of the investigated DLBCL cell lines was quantified upon single treatment with the indicated AZD8186 concentrations after 120 h using the CTG assay. Cell numbers were normalized to the solvent control. Error bars represent standard error of the mean (SEM). **c** DLBCL cell lines were treated with 0.5 µM AZD8186 for 48 h and Annexin V-positive cells were quantified by flow cytometry. The number of Annexin V-positive cells after AZD8186 treatment was normalized to those of DMSO-treated cells. **d** AZD8186 (0.5 µM) induces cell cycle arrest in G0/G1 phase after 24 h in the AZD8186-sensitive cell lines HT, K422, OCI-Ly10, TMD8, and Riva, but not in AZD8186-resistant models OCI-Ly1 and OCI-Ly3. **a**–**d** Representative data from at least three independent experiments are shown. Error bars correspond to the mean ± standard deviation (SD) if not stated otherwise. *P < .05, **P < .01, ***P < .001.

To determine whether the specific PI3Kβ/δ inhibitor AZD8186 potentially represents a promising compound for the treatment of patients with DLBCL, we investigated a panel of 19 DLBCL cell lines of various molecular subtypes. First, we determined the expression status of the PI3Kα, PI3Kβ, and PI3Kδ isoforms, the phosphorylation status of AKT as a marker of pathway activation, as well as expression of PTEN. In the investigated DLBCL cell lines the three PI3K isoforms were expressed to varying degrees and there was no obvious correlation between PI3K expression levels and the DLBCL subtypes or the phosphorylation status of AKT (Fig. 1a). As previously reported, PTEN loss was detectable predominantly in models of GCB DLBCL, which renders them sensitive to AKT inhibition [12, 13].

Next, we treated the cell lines with AZD8186, a PI3K inhibitor that targets preferentially PI3Kβ, but also the PI3Kδ isoform, and determined cell viability after five days [25]. Reduced viability was observed in five out of 19 DLBCL cell lines, including three out of six ABC (OCI-Ly10, TMD8, and Riva) and two out of 13 GCB (HT and K422) DLBCL cell lines suggesting that DLBCLs from both major subtypes can respond to PI3Kβ/δ inhibition (Fig. 1b). All responding cell lines exhibited pathway activation as assessed by phosphorylation of AKT, albeit to varying degree (Fig. 1a). However, not all models characterized by constitutive PI3K/AKT signaling responded to AZD8186, which might implicate that the phosphorylation status of AKT is not sufficient to predict the response to PI3Kβ/δ inhibition. Furthermore, since neither the PI3Kδ-specific inhibitor idelalisib nor the PI3Kα/δ-specific inhibitor AZD8835 could impair the growth of the GCB DLBCL lines HT and K422, our data suggest a so far unappreciated pro-survival role for the PI3Kβ isoform in DLBCL (Supplementary Fig. 1a–b).

To obtain additional insights into the nature of the growth inhibitory effect of AZD8186, we determined the rate of apoptosis and analyzed changes in cell cycle following PI3Kβ/δ inhibition. Apoptosis induction was detectable in the ABC DLBCL cell lines OCI-Ly10, TMD8 and Riva, but not in the GCB DLBCL (HT, K422) or the AZD8186-resistant cells OCI-Ly1 and OCI-Ly3 that served as negative controls (Fig. 1c). In contrast, AZD8186 treatment induced G0/G1 cell cycle arrest in all five sensitive cell lines but not in the two resistant models (Fig. 1d). Taken together, these results suggest that PI3Kβ/δ inhibition interferes with the growth of several DLBCL cell lines characterized by constitutive PI3K/AKT signaling independent of their molecular subtype.

### PI3Kβ/δ inhibition impairs NF-κB signaling in ABC DLBCLs

**Fig. 2:**
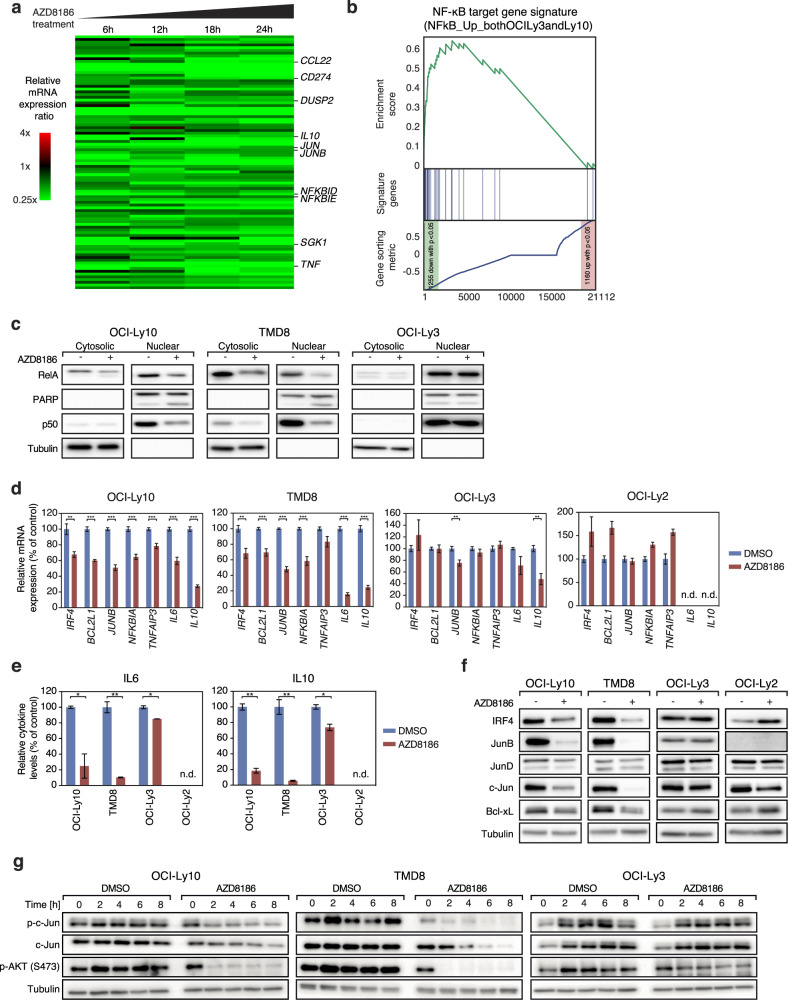
AZD8186 decreases NF-κB and JUN-family signaling in ABC DLBCL. **a** Heatmap of differentially expressed genes in OCI-Ly10 and TMD8 treated with AZD8186 for 6, 12, 18 and 24 h compared to the solvent control. Gene expression changes are depicted according to the color scale. Representative genes that are involved in critical biological processes are highlighted. **b** Gene set enrichment analysis shows a previously described NF-κB gene expression signature, which is significantly enriched with genes that are downregulated after AZD8186 treatment. **c** AZD8186 treatment results in reduced cytoplasmic and nuclear expression of the NF-κB subunits RelA and p50 in AZD8186-sensitive OCI-Ly10 and TMD8 cells, but not in resistant OCI-Ly3 cells. Successful nuclear and cytosolic fractionation is indicated by Tubulin and PARP content. **d** NF-κB target genes are downregulated on mRNA level in OCI-Ly10 and TMD8, but not in OCI-Ly3 and OCI-Ly2 cells after AZD8186 treatment for 24 h. **e** The secretion of IL6 and IL10 was quantified after AZD8186 treatment for 24 h by ELISA and normalized to the solvent control. **f** Protein expression of NF-κB targets IRF4, BCL-xL and JUN-family members (JunB, JunD and c-Jun) was donwregulated after AZD8186 treatment in OCI-Ly10 and TMD8, but not in OCI-Ly3 and OCI-Ly2 cells after 24 h of AZD8186 treatment. **g** c-Jun expression and phosphorylation was visualized after solvent or AZD8186 treatment for the indicated timepoints by immunoblotting. **a**–**g** 0.5 µM AZD8186 was used in the shown experiments. **c**–**g** Data are representative of at least three independent experiments. Error bars correspond to the mean ± SD. n.d., not detectable. *P < .05, **P < .01, ***P < .001.

To understand which biologic processes are affected by PI3Kβ/δ inhibition, we performed gene expression profiling (GEP) using RNA-sequencing in two AZD8186-sensitive ABC DLBCL cell lines (OCI-Ly10 and TMD8) after 6, 12, 18, and 24 h of AZD8186 treatment (Fig. 2a; Supplementary Fig. 2; Supplementary Table 3). An unbiased gene set enrichment analysis (GSEA) using a database of 23026 previously described gene signatures revealed that AZD8186 treatment affected the expression of genes that are enriched in several known NF-κB gene signatures or in a gene signature reflecting changes in expression caused by the PI3Kα/δ inhibitor AZD8835 (Fig. 2b; Supplementary Fig. 3a) [26]. Seven of the top 30 gene sets downregulated upon AZD8186 treatment were NF-κB related signatures and contained several well-known classical NF-κB target genes, such as *CFLAR, NFKBID, NFKBIA, NFKBIE*, and *TNF* (Supplementary Table 4) [12, 27].

To validate if oncogenic NF-κB signaling is indeed downregulated following AZD8186 treatment, we analyzed nuclear RelA and p50 levels in two sensitive (OCI-Ly10 and TMD8) and one resistant (OCI-Ly3) ABC DLBCL cell line. Indeed, nuclear translocation of RelA and p50 but also the total levels of RelA were decreased upon PI3Kβ/δ inhibition in OCI-Ly10 and TMD8, but not in the insensitive OCI-Ly3 cells (Fig. 2c). Additionally, we investigated the expression of various NF-κB targets that mediate survival in ABC DLBCL, such as *IL6*, *IL10*, *IRF4*, and *BCL2L1* on mRNA level by quantitative PCR and on protein level using ELISA and Western blotting (Fig. 2d–f). On transcript level, virtually all mentioned target genes were significantly downregulated after 24 h of AZD8186 treatment in OCI-Ly10 and TMD8 (except for *TNFAIP3* expression in TMD8) whereas in insensitive OCI-Ly3 cells only two genes were moderately downregulated (*JUNB* and *IL10*) and all genes were unaffected in the resistant OCI-Ly2 cells following PI3Kβ/δ inhibition (Fig. 2d). Accordingly, IL6, IL10, IRF4, JunB, c-Jun and Bcl-xL were significantly reduced on protein level following AZD8186 treatment as determined by ELISA and immunoblotting in OCI-Ly10 and TMD8 (Fig. 2e–f). In contrast, none of these proteins were affected in the AZD8186-resistant OCI-Ly2 and OCI-Ly3 (except for a moderate impairment of IL6 and IL10 in OCI-Ly3) by AZD8186 treatment (Fig. 2e–f). Altogether, these data implicate that AZD8186, most likely due to its PI3Kδ inhibitory effect, impairs oncogenic NF-κB activity in ABC DLBCL.

Interestingly, we noticed that AZD8186 significantly decreased the protein expression of the AP-1 members c-Jun and JunB (Fig. 2f). To further decipher the molecular mechanisms underlying the AZD8186-induced downregulation of the AP-1 family members, we analyzed c-Jun Ser63 phosphorylation after AZD8186 treatment as c-Jun protein stability is regulated by SAPK/JNK-mediated phosphorylation. In the AZD8186-sensitive cell lines OCI-Ly10 and TMD8, PI3Kβ/δ inhibition led to decreased c-Jun phosphorylation after two hours and thus preceded the reduction of total c-Jun levels (Fig. 2g). To confirm that PI3Kβ/δ signaling indeed controls c-Jun phosphorylation, we treated OCI-Ly10, TMD8, and OCI-Ly3 cells with the pan-PI3K inhibitors GDC-0941 or LY294002. These analyses confirmed that PI3K inhibitors reduce c-Jun phosphorylation in sensitive but not in the insensitive OCI-Ly3 cells (Supplementary Fig. 3b). At last, to validate that downregulation of c-Jun is controlled by proteasomal degradation, we treated TMD8 cells with AZD8186 as well as with the proteasome inhibitor MG132. Under these conditions, MG132 significantly increased c-Jun expression in AZD8186-treated cells (Supplementary Fig. 3c). Collectively, our results indicate that PI3Kβ/δ inhibition induces toxicity in ABC DLBCL models by impairing the activity of the oncogenic NF-κB and AP-1 transcription factors and thus reducing the expression of pro-survival target genes.

### PI3Kβ/δ inhibition downregulates MYC signaling in GCB DLBCLs

**Fig. 3:**
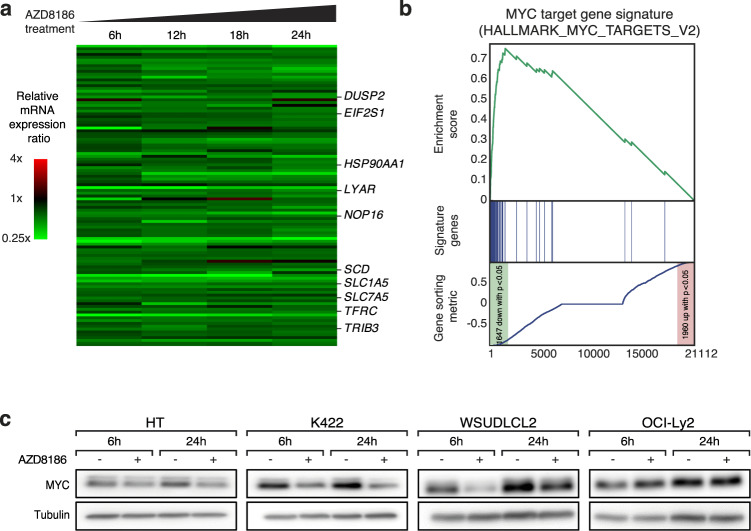
AZD8186 regulates MYC expression in GCB DLBCL. **a** Heatmap of differentially expressed genes in HT and K422 cells treated with AZD8186 for 6, 12, 18 and 24 h compared to the solvent control. Gene expression changes are depicted according to the color scale. Representative genes that are involved in critical biological processes are highlighted. **b** Gene set enrichment analysis shows a previously described MYC expression signature, which is significantly enriched with genes that are downregulated after AZD8186 treatment. **c** The indicated cell lines were treated with 0.5 µM of AZD8186 for 6 and 24 h and MYC protein expression was analyzed by immunoblotting. Tubulin served as loading control. Data are representative of at least three independent experiments.

To investigate the molecular mechanisms underlying the toxicity of PI3Kβ/δ inhibition in GCB DLBCL models, we performed GEP after 6, 12, 18, and 24 h of AZD8186 treatment in the two sensitive GCB DBLCL cell lines HT and K422 (Fig. 3a; Supplementary Fig. 4; Supplementary Table 5). The GSEA identified multiple previously described MYC target gene signatures as significantly downregulated after AZD8186 treatment (Fig. 3b; Supplementary Fig. 5a). Among the top 30 downregulated gene sets following AZD8186 treatment, five were MYC-related signatures suggesting that deregulation of the gene expression network of MYC is a central mechanism of action of PI3Kβ/δ inhibition (Supplementary Table 6). To determine the expression of MYC following PI3Kβ/δ inhibition, we treated two AZD8186-sensitive (HT and K422) and two insensitive (WSUDLCL2 and OCI-Ly2) GCB DLBCL models with AZD8186 and visualized MYC protein levels by immunoblotting. Indeed, MYC protein levels were reduced in the AZD8186-sensitive cell lines HT and K422 (Fig. 3c). In contrast, MYC levels were unaffected in AZD8186-resistant OCI-Ly2 cells, whereas in insensitive WSUDLCL2 cells an initial drop of MYC protein levels after 6 h could be detected, which recovered after 24 h of treatment (Fig. 3c). To investigate the importance of MYC downregulation by AZD8186 for cell survival, we exogenously expressed MYC in the GCB DLBCL cell line HT and monitored its response to PI3Kβ/δ inhibition. MYC expression mediated resistance to AZD8186 treatment, indicating an essential role of the transcription factor for the survival-promoting effect of the PI3K signaling pathway in GCB DLBCL (Supplementary Fig. 5b).

Collectively, these data indicate that PI3Kβ/δ signaling regulates MYC expression in GCB DLBCLs, which might contribute to the G0/G1 cell cycle arrest observed upon AZD8186 treatment (Fig. 1d) [28].

### PI3Kα mediates resistance to AZD8186 treatment

**Fig. 4:**
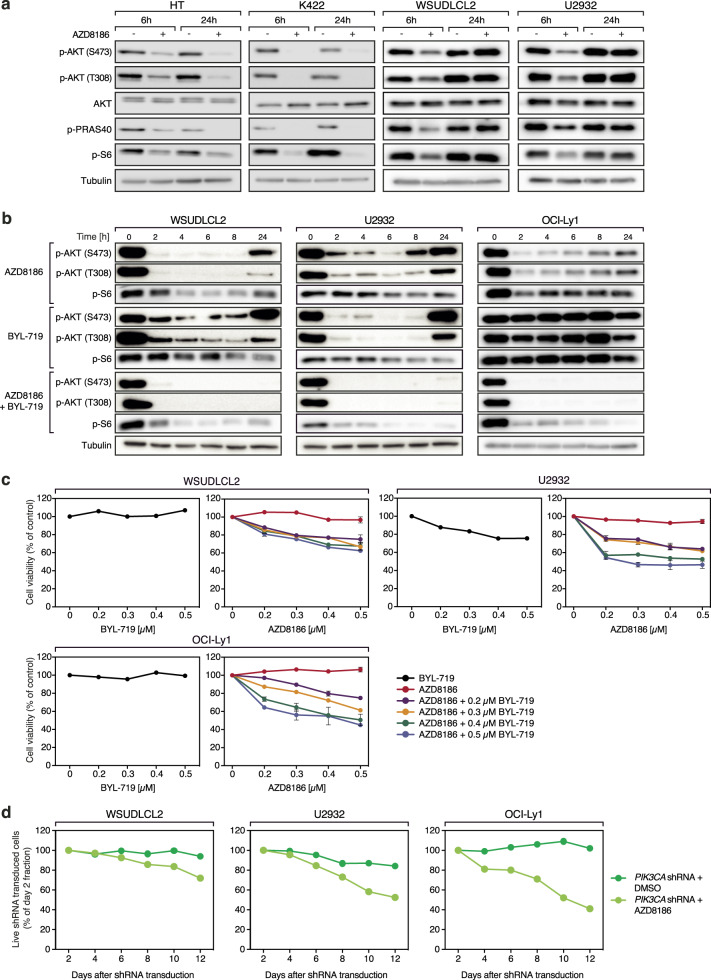
PI3Kα activity mediates resistance to AZD8186. **a** AZD8186-sensitive (HT, K422) and -resistant (WSUDLCL2, U2932) DLBCL cell lines were treated for 6 and 24 h with AZD8186 and respective lysates were analyzed for the phosphorylation of AKT, PRAS40 and S6 by immunoblotting. **b** The indicated DLBCL cell lines were treated either with AZD8186, BYL-719 or with the combination of both inhibitors and the phosphorylation status of AKT and S6 was visualized by immunoblotting. **c** Combined AZD8186 and BYL-719 treatment for 120 h induces synergistic cytotoxicity in single treatment-resistant DLBCL cells, as measured by CTG assay. Left panel shows DMSO-normalized BYL-719 treatment, right panel represents BYL-719-normalized combination treatments with AZD8186 as indicated. **d** The indicated DLBCL cell lines were transduced with a vector carrying a *PIK3CA*-specific shRNA and GFP. The proportion of GFP-positive cells was quantified after treatment with solvent or AZD8186 by flow cytometry and normalized to the control shRNA. Concentrations of inhibitors used in these experiments are as following: 0.5 µM of AZD8186 (**a**–**d**) and 0.5 µM of BYL-719 (**b**, **c**). For all experiments, the representative data of at least three independent experiments are shown. Error bars indicate SD.

To investigate why some DLBCL cell lines are resistant against AZD8186, we monitored the activation of the PI3K-dependent phosphorylation of AKT and ribosomal protein S6 (S6) over time. Interestingly, AKT and S6 phosphorylation were initially impaired after AZD8186 treatment in all investigated DLBCL cell lines, but recovered within 24 h only in the resistant DLBCL cell lines WSUDLCL2 and U2932 (Fig. 4a). In line, the phosphorylation of PRAS40, which is part of the downstream mTOR complex 1 (mTORC1), was reduced in all cell lines, but recovered in the resistant DLBCL cell lines 24 h after treatment (Fig. 4a), suggesting that the resistant cells compensated for the lack of PI3Kβ/δ activity by an alternative way of S6 and PRAS40 activation.

As there is evidence for a functional redundancy between the PI3K isoforms, and, at least in a *PTEN*-deficient background, PI3Kα can compensate for the loss of PI3Kβ in solid tumors, we investigated the importance of PI3Kα signaling in DLBCL models resistant to AZD8186 [29–31]. Similar to the single treatment with AZD8186, the PI3Kα specific inhibitor BYL-719 led to an initial decrease in the phosphorylation of AKT in the AZD8186-resistant DLBCL cell lines WSUDLCL2, and U2932 (Fig. 4b). In contrast, in OCI-Ly1 cells AZD8186 treatment initially decreased AKT phosphorylation levels while BYL-719 treatment did not affect AKT activation (Fig. 4b). Interestingly, neither AZD8186 nor BYL-719 had the potential to suppress the activity of AKT for more than 24 h in the resistant cell lines (Fig. 4b). However, the simultaneous block of PI3Kβ/δ and PI3Kα by co-treatment of AZD8186 and BYL-719 prevented the reactivation of the signaling pathway after 24 h in the AZD8186-resistant DLBCL cell lines, suggesting a potential feedback activation by PI3Kα (Fig. 4b). To further explore whether the inhibition of PI3Kα could sensitize AZD8186-resistant DLBCL cell lines towards the PI3Kβ/δ inhibitor, we treated OCI-Ly1, WSUDLCL2 and U2932 cells with BYL-719 alone or in combination with AZD8186. Notably, the AZD8186-resistant DLBCL cell lines were also not sensitive to BYL-719 single treatment, but the combination of both inhibitors synergistically reduced cell growth of all cell lines (Fig. 4c; Supplementary Fig. 6). To confirm the role of PI3Kα in AZD8186 resistance and to avoid misinterpretation due to off-target effects of the inhibitors, we used an shRNA-based approach to silence *PIK3CA*, which encodes for PI3Kα, and additionally inhibited PI3Kβ/δ using AZD8186. Indeed, silencing of *PIK3CA* induced sensitivity to AZD8186 in all resistant DLBCL models (Fig. 4d; Supplementary Fig. 7a). Vice versa, *PIK3CB* (coding for PI3Kβ) silencing rendered the cells susceptible to the PI3Kα/δ-specific inhibitor AZD8835, suggesting that at least the PI3Kα and β isoforms have redundant molecular functions and that for some DLBCLs all three isoforms need to be targeted to induce cytotoxicity (Supplementary Fig. 7b–c).

### Dual PI3Kβ/δ and mTOR inhibition is highly effective in DLBCLs both in vitro and in vivo

**Fig. 5:**
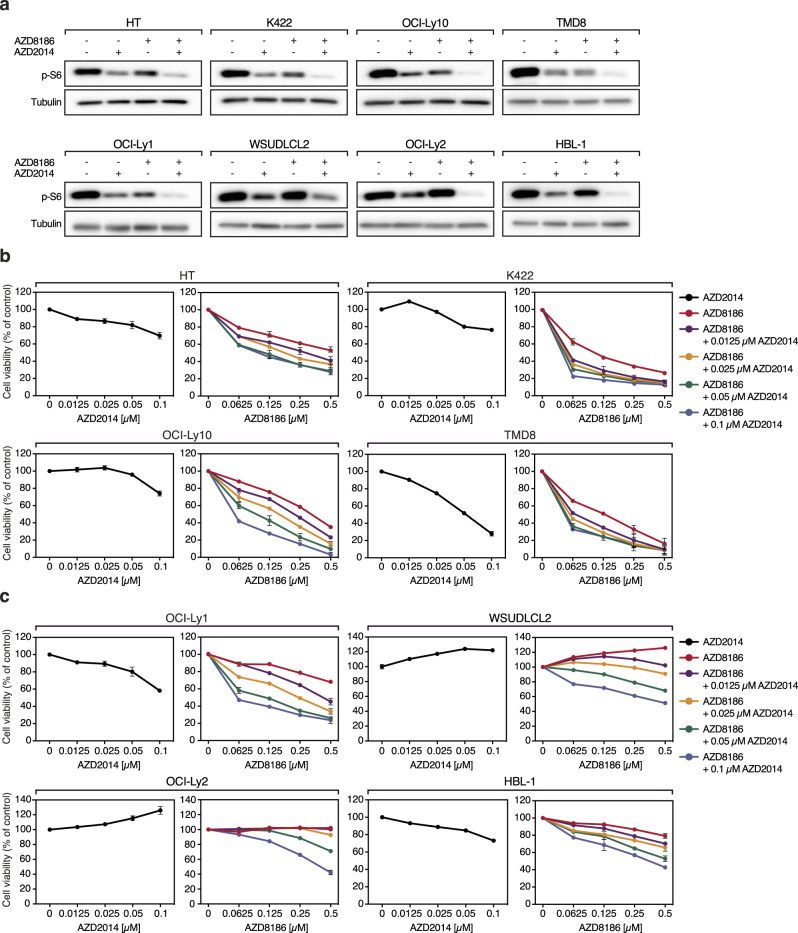
AZD8186 and AZD2014 synergistically impair the growth of DLBCL cells in vitro. **a** S6 phosphorylation was analyzed in AZD8186-sensitive (HT, K422, OCI-Ly10 and TMD8) and -resistant cell lines (OCI-Ly1, WSUDLCL2, OCI-Ly2, and HBL-1) that were either treated with AZD8186 (0.5 µM), AZD2014 (0.1 µM) or with the combination of both inhibitors for 24 h. **b, c** AZD8186-sensitive (**b**) or -resistant (**c**) DLBCL cell lines were treated for 120 h with the indicated AZD8186 and AZD2014 concentrations and cell viability was determined. For each cell line, left panel shows DMSO-normalized AZD2014 treatment, right panel represents AZD2014-normalized combination treatments with AZD8186. Data are representative of at least three independent experiments. Error bars correspond to the mean ± SD.

Since mTOR inhibitors are in general well tolerated and demonstrated at least partial efficacy in relapsed DLBCL patients, we investigated whether mTOR inhibition is sufficient to prevent the reactivation of S6 phosphorylation in AZD8186-treated DLBCL cell lines [32–34]. Whereas neither AZD8186 nor the ATP-competitive mTORC1/2 inhibitor AZD2014 (vistusertib) were able to suppress S6 phosphorylation completely, the combination of both inhibitors strongly reduced S6 activation in AZD8186-sensitive and -resistant DLBCL cell lines (Fig. 5a). Accordingly, the combinatorial treatment with AZD8186 and AZD2014 synergistically impaired growth of AZD8186-sensitive cell lines (Fig. 5b; Supplementary Fig. 8). More importantly, we noticed that AZD2014 treatment was able to sensitize previously AZD8186-resistant DLBCL cells to PI3Kβ/δ inhibition, confirming the importance of PI3K/AKT/mTOR reactivation as a resistance mechanism in DLBCL (Fig. 5c; Supplementary Fig. 9). Single silencing of either PI3Kβ or PI3Kδ expression alone was not sufficient to promote AZD2014-induced toxicity in OCI-Ly1 cells, only the combined silencing of both isoforms potentiated the effect of AZD2014 (Supplementary Fig. 10a). In contrast, targeting mTORC1 and not mTORC2 using temsirolimus showed comparable capacity to enhance AZD8186-mediated killing similar to the mTORC1/2 inhibitor AZD2014 (Supplementary Fig. 10b). Only one cell line, OCI-Ly3, which harbors the activating *CARD11*^L244P^ mutation, did not respond to this combination treatment (Supplementary Fig. 11a, b). We thus hypothesized that the constitutive activation of CARD11 compensated for the lack of PI3Kβ/δ and mTOR signaling. To test this hypothesis, we dampened CARD11-mediated signaling by the inhibition of its downstream effector MALT1 [35]. Indeed, MALT1 inhibition rendered OCI-Ly3 susceptible to AZD8186 treatment, highlighting the potential of oncogenic CARD11 to mediate resistance against the dual inhibition of PI3Kβ/δ and mTOR (Supplementary Fig. 11c, d).

**Fig. 6:**
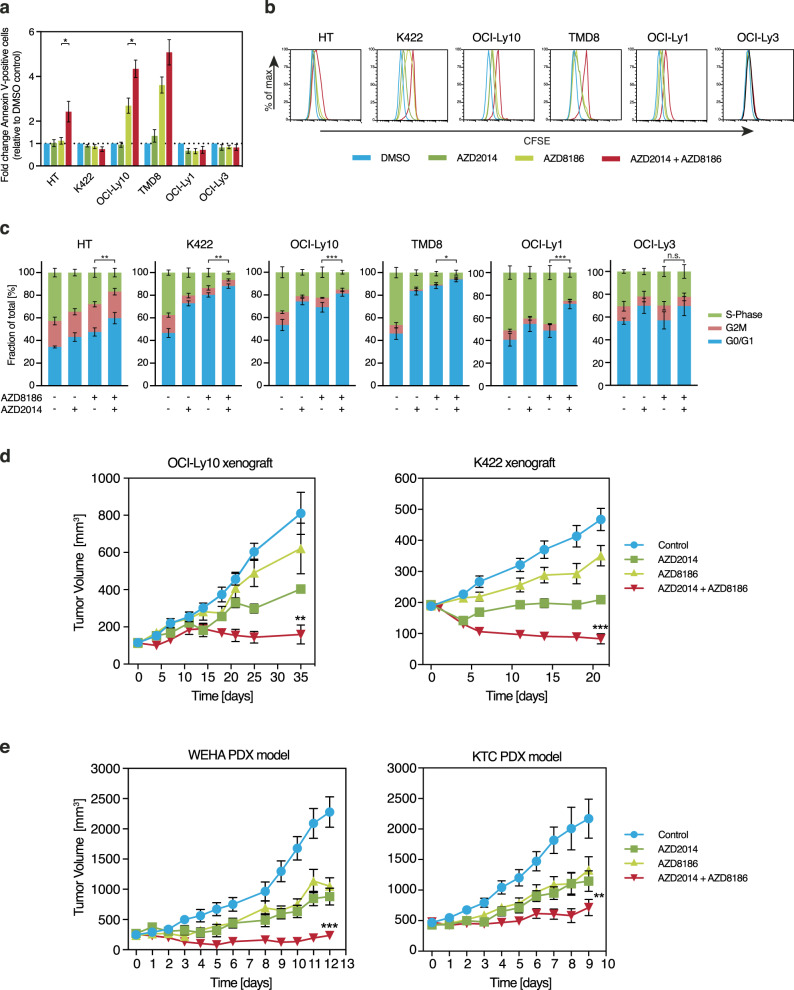
AZD8186 and AZD2014 exhibit synergistic effects in vivo. **a**–**c** DLBCL cell lines were treated either with solvent, AZD8186 (0.5 µM), AZD2014 (0.1 µM) or AZD8186 in combination with AZD2014 as indicated. **a** After 48 h, Annexin V-positive cells were quantified by flow cytometry and normalized to DMSO-treated controls. **b** CFSE dilutions were measured at 120 h of treatment by flow cytometry. Representative results of CFSE intensities are shown. **c** To assess the frequency of cells in different cell cycle phases, DNA was stained by DAPI and quantified by image analysis after 24 h of treatment. **d, e** Tumor growth curves of xenograft mouse models following treatment with vehicle control (blue), AZD2014 15 mg/kg (dark green), AZD8186 50 mg/kg (light green), and AZD2014 + AZD8186 (red). Treatment was initiated after animals developed macroscopic signs of tumors. In the OCI-Ly10- and K422-based mouse xenograft models (**d**), the combinatorial treatment significantly reduced tumor growth in comparison to single treatments (*P* = .0027 for AZD8186 [*n* = 10] vs. AZD8186/AZD2014 [*n* = 10] on day 35 for OCI-Ly10, *P* = 4.6 × 10^-5^ for AZD8186 [*n* = 9] vs. AZD8186/AZD2014 [*n* = 5] on day 20 for K422). **e** The combination of AZD8186 and AZD2014 significantly impaired tumor growth in the two independent WEHA and KTC PDX mouse models compared to AZD8186 treatment alone (*P* = .0006 for AZD8186 [*n* = 7] vs. AZD8186/AZD2014 [*n* = 7] on day 12 for WEHA, *P* = .002 for AZD8186 [*n* = 8] vs. AZD8186/AZD2014 [*n* = 8] on day 9 for KTC). Error bars indicate SEM. *P < .05, **P < .01, ***P < .001.

In comparison to the single inhibitor treatments, the combination of AZD8186 and AZD2014 resulted in increased apoptosis induction or impaired proliferation in the majority of DLBCL cell lines (Fig. 6a, b; Supplementary Fig. 12a, b). Image analysis of the fluorescently stained cellular DNA content suggested that the reduction in cell proliferation was caused by a G0/G1 cell cycle block (Fig. 6c; Supplementary Fig. 12c).

To evaluate the anti-tumor effect of simultaneous inhibition of PI3Kβ/δ and mTOR in vivo, we co-administrated AZD8186 and AZD2014 in an ABC (OCI-Ly10) and a GCB (K422) DLBCL xenograft mouse model. Whereas the respective single treatments were only able to slow down tumor growth in these xenograft mouse models, the combination treatment completely stabilized (OCI-Ly10) or significantly reduced (K422) the tumor size (*P* = .0027 for AZD8186 vs. AZD8186/AZD2014 on day 35 for OCI-Ly10, *P* = 4.6 × 10^-5^ for AZD8186 vs. AZD8186/AZD2014 on day 20 for K422) (Fig. 6d). To confirm these strong anti-lymphoma effects of the combined AZD8186 and AZD2014 treatment in a setting of relapsed/refractory DLBCL patients, we used two independent patient-derived xenograft (PDX) mouse models that were derived from refractory DLBCL patients. The WEHA model was generated using cells from a refractory GCB DLBCL, the KTC from a refractory non-GCB DLBCL patient. Interestingly, even in the refractory PDX models, the simultaneous PI3Kβ/δ and mTOR inhibition resulted in a significant synergistic effect compared to the single drugs alone which prevented the outgrowth of the tumor (*P* = .0006 for AZD8186 vs. AZD8186/AZD2014 on day 12 for WEHA, *P* = .002 for AZD8186 vs. AZD8186/AZD2014 on day 9 for KTC) (Fig. 6e). We detected no weight loss in the single and the dual AZD8186 and AZD2014 treated xenograft and PDX models, suggesting that the inhibitors did not provoke severe adverse side effects (Supplementary Fig. 13a, b). Taken together, these results indicate that the simultaneous pharmacological inhibition of PI3Kβ/δ and mTOR has a broad and profound anti-lymphoma activity and is able to abolish DLBCL tumor growth in vivo.

## Discussion

In the present study, we demonstrate for the first time that a subset of DLBCL models is addicted to PI3Kβ/δ signaling. Whereas dual inhibition of PI3Kα/δ has been shown to mainly affect the NF-κB-dependent ABC DLBCL subtype, we are unable to correlate the efficacy of PI3Kβ/δ inhibition with any known molecular DLBCL subtype [12]. In solid tumors, previous studies have suggested that the loss of PTEN correlates with the susceptibility towards PI3Kβ inhibition [25, 36–39]. In contrast, our current work demonstrates that the PI3Kβ/δ inhibitor AZD8186 exhibits activity in both PTEN-expressing and PTEN-deficient DLBCL models. In AZD8186-sensitive cell lines, PI3Kβ/δ inhibition impaired NF-κB signaling in ABC DLBCL, while reducing expression of the oncogenic transcription factor MYC in GCB DLBCLs. These effects most likely mediate the growth-inhibiting effect of AZD8186 in the respective DLBCL subset. How NF-κB signaling in ABC DLBCL is impaired by PI3Kβ/δ blockade is not well understood, but direct activation of the IKK complex by AKT has been proposed [40, 41]. Furthermore, we also detected for the first time that PI3Kβ/δ signaling regulates c-Jun expression both on a transcriptional and posttranslational level in ABC DLBCL. Jun signaling has been shown to contribute to lymphoma growth and dissemination to extra-nodal sites, highlighting the potential of PI3K inhibitors to impede several key pathways important for DLBCL survival and dissemination [42].

Whereas PI3Kβ/δ inhibition resulted in an initial reduction of the activity of the AKT/mTOR/S6 axis in the majority of the DLBCLs, we observed a reactivation of the signaling pathway after 24 h in the AZD8186-resistant DLBCL models. Since the simultaneous blockade of PI3Kα or mTOR prevented this rebound, we propose a mechanism in which PI3Kβ/δ inhibition promotes a PI3Kα-mediated mTOR activation [43]. How PI3Kα is activated in PI3Kβ/δ inhibited DLBCL cells and why only a selection of DLBCL cell lines can escape PI3Kβ/δ inhibition remain unclear. Resistance against the PI3Kδ-specific inhibitor idelalisib has been correlated with increased phosphorylation of BCAP and CD19 or CXCR4 upregulation, driving the reactivation of the PI3K pathway [24, 44, 45]. A similar model has been proposed in *PTEN*-mutated solid cancer cells, in which PI3Kβ inhibition leads to IGF1R-dependent reactivation of PI3Kα [31].

To circumvent potential resistance by upregulation/reactivation of distinct PI3K isoforms, the use of a pan-PI3K inhibitor would seem the logical choice for anti-lymphoma treatment. However, the respective drugs experienced a setback due to the lack of activity and safety concerns [46]. Thus, we propose a combination of an isoform-specific PI3Kβ/δ inhibitor with an mTORC1/2 inhibitor, especially since this combination did not lead to a treatment-associated weight loss in treated mice and resulted in a stabilization/regression of the tumors in the cell line- and patient-derived DLBCL xenograft models. The monotherapy with mTORC1 or mTORC1/2 inhibitors is generally well tolerated by patients, but exhibits only a limited efficacy with approximately 30% of patients responding to treatment [34, 47, 48]. Our results suggest simultaneous PI3Kβ/δ blockade would not only markedly increase the efficacy of mTOR inhibitors but would also sensitize mTOR or PI3Kβ/δ inhibitor-resistant cells to the treatment, independent of their DLBCL classification. Strikingly, the combinatorial treatment also showed activity against cells originating from refractory GCB and non-GCB DLBCL patients, indicating that dual mTOR and PI3Kβ/δ inhibition might represent a promising novel strategy to target tumor cells resistant to first-line therapy.

The only DLBCL model investigated which neither responded to AZD8186 monotreatment nor to the combination with AZD2014, was OCI-Ly3, which harbors the *CARD11*^L244P^ gain-of-function mutation [49]. Since a MALT1 inhibitor was able to sensitize OCI-Ly3 cells to AZD8186, we propose that constitutively active CARD11 is able to overwrite the effects of impaired PI3K/mTOR signaling due to a strong activation of NF-κB and c-Jun [49, 50].

Collectively, we demonstrate for the first time that a subset of DLBCL models is addicted to PI3Kβ/δ signaling. Comprehensive pathway inhibition preventing feedback mediated resistance can be overcome by the combination with the mTOR inhibitor AZD2014 in different molecular subtypes of DLBCL. Thus, our data provide a strong rationale to combine PI3Kβ/δ with mTOR inhibitors in future clinical trials.

## Supplementary information

Revised Supplementary Information

Supplementary Table 3

Supplementary Table 4

Supplementary Table 5

Supplementary Table 6

## Data Availability

The data reported in this article have been deposited in the Gene Expression Omnibus database (accession number GSE212746).
