# Supplementary material for: mTOR inhibition amplifies the anti-lymphoma effect of PI3Kβ/δ blockage in diffuse large B-cell lymphoma
Source: Leukemia. 2022 Nov 9;37(1):178–89. doi: 10.1038/s41375-022-01749-0 (PMC9883168; doi:10.1038/s41375-022-01749-0)
Supplement: Supplementary file 1 — Revised Supplementary Information [file 41375_2022_1749_MOESM1_ESM.pdf]

Supplementary Information for

mTOR inhibition amplifies the anti-lymphoma effect of PI3Kβ/δ blockage in diffuse large B-cell lymphoma

Wendan Xu[1*], Philipp Berning[1*], Tabea Erdmann[1*], Michael Grau[1], Nardjas Bettazová[2,3], Myroslav Zapukhlyak[1], Fabian Frontzek[1], Corinna Kosnopfel[1], Peter Lenz[4,5], Michael Grondine[6], Brandon Willis[6], James T. Lynch[7], Pavel Klener[2,8], Stephan Hailfinger[1], Simon T. Barry[7], Georg Lenz[1]

[1]Department of Hematology, Oncology and Pneumology, University Hospital Muenster, Muenster, Germany
[2]Institute of Pathological Physiology, First Faculty of Medicine, Charles University, Prague, Czech Republic
[3]Department of Medical Genetics, Third Faculty of Medicine, Charles University, Prague, Czech Republic
[4]Department of Physics, University of Marburg, Marburg, Germany
[5]LOEWE Center for Synthetic Microbiology, Marburg, Germany
[6]Bioscience, Early Oncology, AstraZeneca, Boston, MA, USA
[7]Bioscience, Early Oncology, AstraZeneca, Cambridge, United Kingdom
[8]First Department of Internal Medicine - Department of Hematology, University General Hospital and First Faculty of Medicine, Charles University, Prague, Czech Republic
* Contributed equally to this work.

Corresponding author:
Georg Lenz, MD
University Hospital Münster
Department of Medicine A for Hematology, Oncology and Pneumology
Albert-Schweitzer-Campus 1
48149 Münster
Germany
Email: georg.lenz@ukmuenster.de

mTOR inhibition amplifies the anti-lymphoma effect of PI3Kβ/δ blockage in diffuse large B-cell lymphoma

Supplementary Materials and Methods

Cell culture, retroviral constructs, and transductions

Human DLBCL cell lines were cultured in RPMI 1640 (Invitrogen) with 10% or 20% fetal calf serum (FCS) except for OCI-Ly3, OCI-Ly10, TMD8, OCI-Ly1, OCI-Ly2, OCI-Ly19 which were cultured in Iscove's modified Dulbecco's medium supplemented with either 10% FCS or 20% human plasma as previously described (1). All cell lines were maintained at 37°C with 5% CO_2. Short tandem repeat (STR) profiling was performed to confirm cell identities and cells were regularly tested to be free of mycoplasma contamination.

For efficient retroviral transductions, all cell lines were engineered to express a murine ecotropic receptor as previously described (2). Additionally, the cell lines were also engineered to express a bacterial tetracycline repressor allowing doxycycline-inducible small hairpin RNA (shRNA) expression. The retroviral transduction experiments, shRNA-mediated RNA interference and cytotoxicity assays were performed as described elsewhere (2-6). In brief, to assess toxicity of an shRNA, retroviruses that co-express green fluorescent protein (GFP) were used as described (2, 4). Flow cytometry was performed two days after shRNA transduction to determine the initial GFP-positive proportion of live cells for each shRNA. Subsequently, cells were cultured with doxycycline (40 ng/mL) to induce shRNA expression and the proportion of GFP-positive cells was measured at indicated time points. In parallel, cells were treated with DMSO or 0.5 µM AZD8186 or 0.8 µM AZD8835. The GFP-positive proportion at each time point was normalized

to that of the negative control shRNA and further normalized to the day two fraction. The targeting sequence of the utilized shRNAs directed against *PIK3CA*, *PIK3CB* and *PIK3CD* are summarized in Supplementary Table 1. As a negative control shRNA, a previously described shRNA against *MSMO1* (**Supplementary Table 1**) was used (4). Each shRNA experiment was completely reproduced at least three times. For the AZD8186 rescue experiment, cells were engineered to express a *MYC* cDNA (NM_002467.4). The AZD8186 rescue experiment was completely reproduced at least three times.

Gene expression profiling

Gene expression profiling was performed in OCI-Ly10, TMD8, HT, and K422 cells after 6, 12, 18 and 24 hours following treatment with 0.5 µM AZD8186 as previously described (3, 7). Total RNA was isolated using the NucleoSpin RNA Plus kit (Macherey-Nagel, Germany) according to the manufacturer's protocol and RNA sequencing was performed. Sequenced reads of mRNA were aligned against the human transcriptome using HISAT2 (8). Aligned sequence counts were then aggregated for genes using RSEM (9). A non-negative binomial test (Bioinformatics Toolbox of MATLAB® R2020a, The MathWorks® Inc., Natick, Massachusetts, USA) was used to calculate *P*-values of gene regulation comparing AZD8186 and DMSO over all time points. The Benjamini and Hochberg method was used to correct for multiple hypothesis testing and compute false discovery rates (FDRs) (10).

To analyze in an unbiased fashion which biological processes were affected by AZD8186, we performed gene set enrichment analysis (GSEA) (11), testing an integrated database of 23026 gene expression signatures for enrichment with respect to the gene ranking by AZD8186 regulation. This database contained

signatures from the Molecular Signatures Database v7.1 (12), the Staudt laboratory library (13), unsupervised SDCM (14) signatures of gene expression heterogeneity in DLBCL, and signatures from our previous papers. GSEA *P*-values were computed by permutation tests and FDRs were computed relative to respective signature families. Signatures with ≤8 defined gene members were excluded.

Quantitative PCR

Total RNA was extracted using the NucleoSpin RNA Plus kit (Macherey-Nagel, Germany) and transcribed into cDNA using High-Capacity cDNA Reverse Transcription Kit with RNase Inhibitor (Applied Biosystems, Waltham, MA, USA). Quantitative PCR was performed using predesigned TaqMan gene expression assays according to the manufacturer’s instructions (Applied Biosystems, Waltham, MA, USA) as reported previously (6).

Western blotting

Western blotting was performed as previously described (4). Nuclear/cytoplasmic extracts were prepared using the Nuclear Extract Kit (Active Motif, Carlsbad, CA, USA) according to the instructions of the manufacturer. All antibodies used in this study are summarized in **Supplementary Table 2**.

***In vivo* xenograft mouse studies**

For the testing of *in vivo* models, six- to eight-week-old female NOD.Cg-*Prkdc* severe combined immunodeficiency *Il2rg*^{tm1Wjl}/SzJ (NSG; Jackson Laboratory, Bar Harbor, ME, USA) mice were used. Once the mice developed macroscopic signs of

subcutaneous tumors, animals were randomized into control, AZD2014 (q.i.d., p.o., 15 mg/kg), AZD8186 (b.i.d., p.o., 50 mg/kg), or AZD2014+AZD8186 treated groups. No blinding was applied. Both inhibitors were formulated in 0.5% HPMC/0.1% Tween 80. Tumor size was measured daily in three perpendicular dimensions for each mouse using caliper. Tumor volume was calculated according to the following formula: $V = \pi/6 \times (\text{length} \times \text{width} \times \text{height})$. All animal experiments were approved by the institutional Animal Care and Use Committee, as well as by the Research and Higher Education section of the Ministry of Education, Youth and Sports of the Czech Republic under the number MSMT-37957/2020-3. Significance was calculated with a one-tailed two-sample t-test.

Synergy analyses

The synergy between inhibitors was computed using the Chou combination index (CI) model (15). Mean CI (MCI) for selected concentration tuples was used to assess synergistic effect (1 = additive; >1 antagonism; <1 synergy). For drug-resistant cell lines for which an IC50 could not be calculated, a concentration of five times the maximum applied single dosage was considered as an IC50 estimate in further synergy CI computation. The slope of the curve is set to the slowest decline under the condition that the viability does not decrease below 99% in the measured range, therefore it still fits all observed values precisely given the inter-replicate variance. Under this assumption, synergies for combinations with such insensitive drugs can still be estimated as lower bounds. However, synergies predicted based on this extrapolation assumption should be interpreted with care and experimentally validated before further usage.

References

1. Pfeifer M, Grau M, Lenze D, Wenzel SS, Wolf A, Wollert-Wulf B, et al. PTEN loss defines a PI3K/AKT pathway-dependent germinal center subtype of diffuse large B-cell lymphoma. Proceedings of the National Academy of Sciences of the United States of America. 2013;110(30):12420-5.
2. Ngo VN, Davis RE, Lamy L, Yu X, Zhao H, Lenz G, et al. A loss-of-function RNA interference screen for molecular targets in cancer. Nature. 2006;441(7089):106-10.
3. Dai B, Grau M, Juilland M, Klener P, Horing E, Molinsky J, et al. B-cell receptor-driven MALT1 activity regulates MYC signaling in mantle cell lymphoma. Blood. 2017;129(3):333-46.
4. Wenzel SS, Grau M, Mavis C, Hailfinger S, Wolf A, Madle H, et al. MCL1 is deregulated in subgroups of diffuse large B-cell lymphoma. Leukemia. 2013;27(6):1381-90.
5. Pfeifer M, Zheng B, Erdmann T, Koeppen H, McCord R, Grau M, et al. Anti-CD22 and anti-CD79B antibody drug conjugates are active in different molecular diffuse large B-cell lymphoma subtypes. Leukemia. 2015;29(7):1578-86.
6. Nogai H, Wenzel SS, Hailfinger S, Grau M, Kaergel E, Seitz V, et al. IkappaB-zeta controls the constitutive NF-kappaB target gene network and survival of ABC DLBCL. Blood. 2013;122(13):2242-50.
7. Erdmann T, Klener P, Lynch JT, Grau M, Vockova P, Molinsky J, et al. Sensitivity to PI3K and AKT inhibitors is mediated by divergent molecular mechanisms in subtypes of DLBCL. Blood. 2017;130(3):310-22.
8. Kim D, Langmead B, Salzberg SL. HISAT: a fast spliced aligner with low memory requirements. Nat Methods. 2015;12(4):357-60.
9. Li B, Dewey CN. RSEM: accurate transcript quantification from RNA-Seq data with or without a reference genome. BMC Bioinformatics. 2011;12:323.
10. Benjamini Y, Hochberg Y. Controlling the False Discovery Rate: A Practical and Powerful Approach to Multiple Testing. Journal of the Royal Statistical Society: Series B (Methodological). 1995;57(1):289-300.
11. Subramanian A, Tamayo P, Mootha VK, Mukherjee S, Ebert BL, Gillette MA, et al. Gene set enrichment analysis: a knowledge-based approach for interpreting genome-wide expression profiles. Proceedings of the National Academy of Sciences of the United States of America. 2005;102(43):15545-50.
12. Liberzon A, Birger C, Thorvaldsdottir H, Ghandi M, Mesirov JP, Tamayo P. The Molecular Signatures Database (MSigDB) hallmark gene set collection. Cell Syst. 2015;1(6):417-25.
13. Shaffer AL, Wright G, Yang L, Powell J, Ngo V, Lamy L, et al. A library of gene expression signatures to illuminate normal and pathological lymphoid biology. Immunol Rev. 2006;210:67-85.
14. Grau M, Lenz G, Lenz P. Dissection of gene expression datasets into clinically relevant interaction signatures via high-dimensional correlation maximization. Nat Commun. 2019;10(1):5417.
15. Chou TC. Theoretical basis, experimental design, and computerized simulation of synergism and antagonism in drug combination studies. Pharmacol Rev. 2006;58(3):621-81.

Xu et al. Supplementary Fig. 1

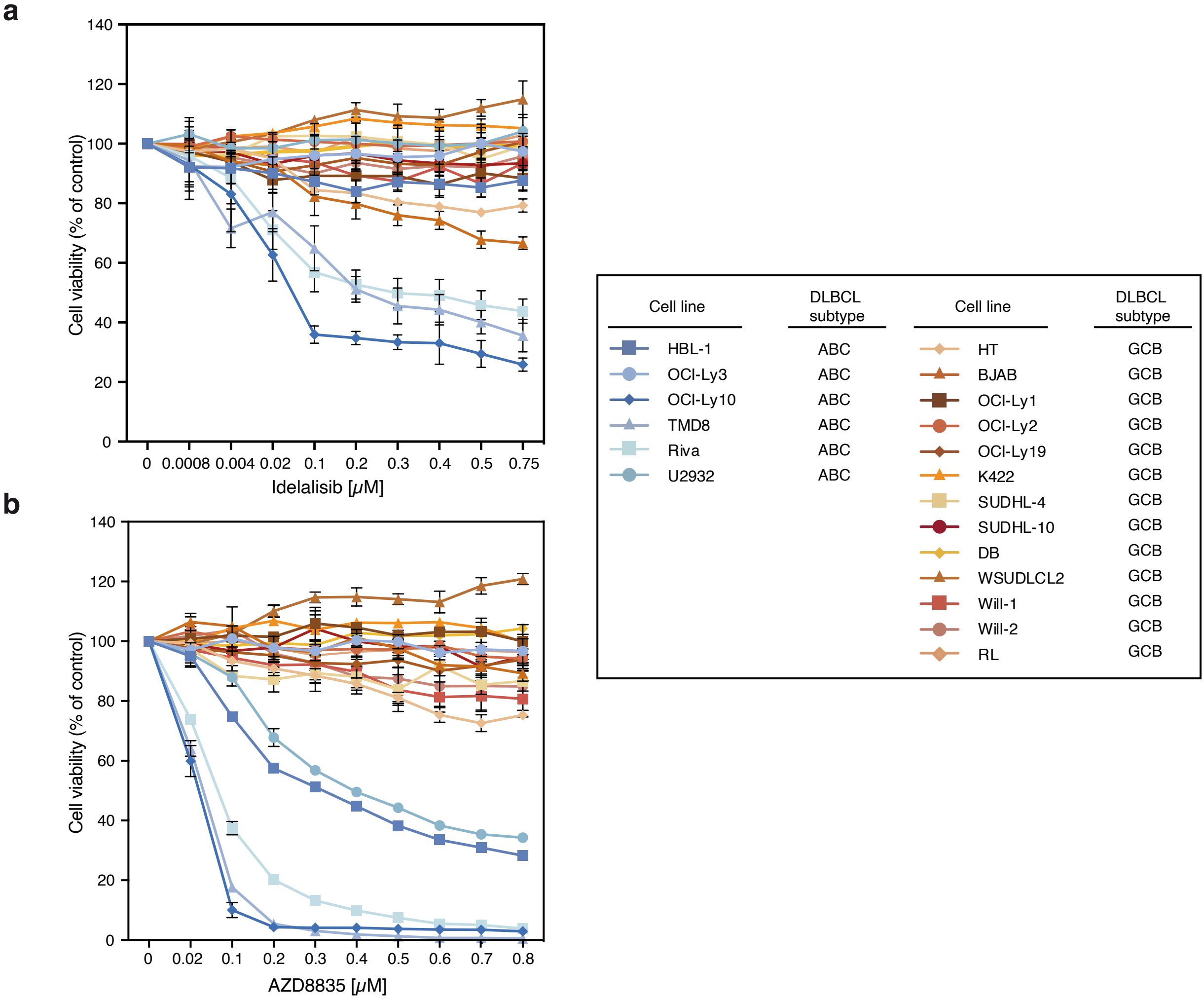

Xu et al. Supplementary Fig. 2

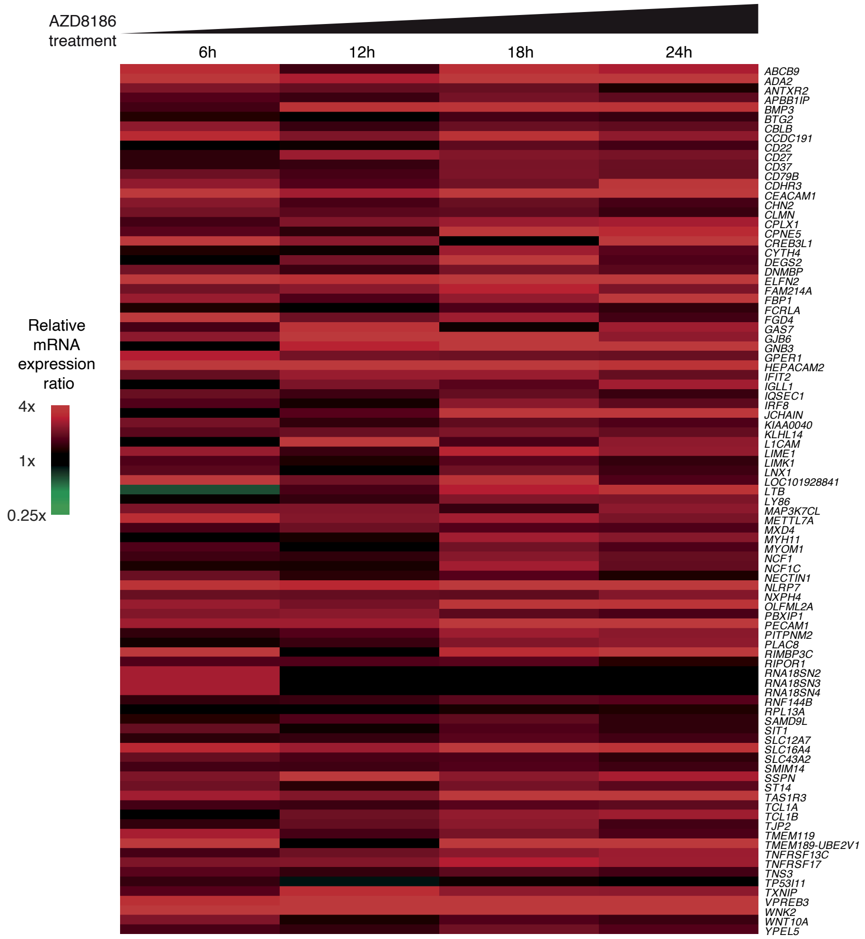

Xu et al. Supplementary Fig. 3

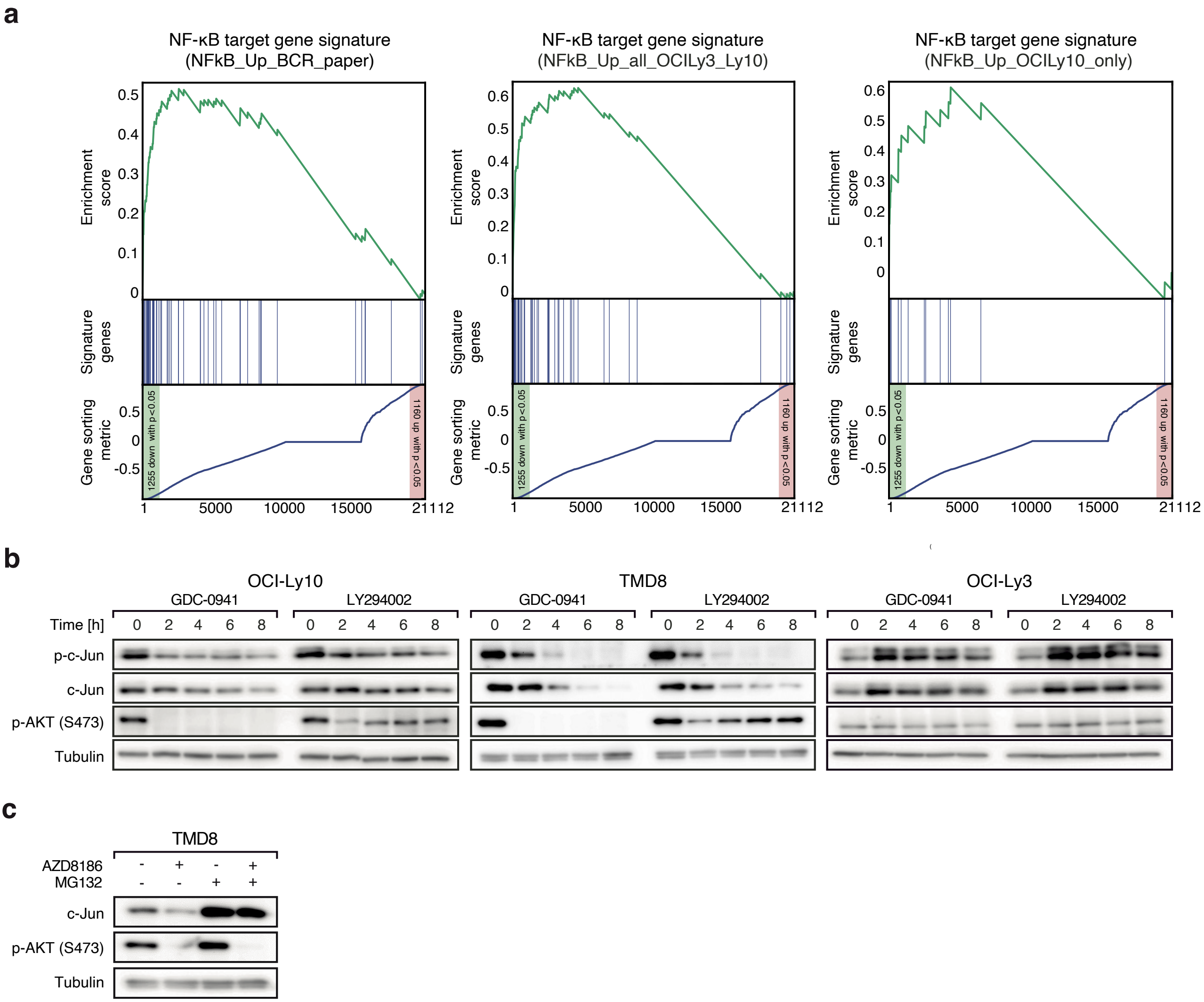

Xu et al. Supplementary Fig. 4

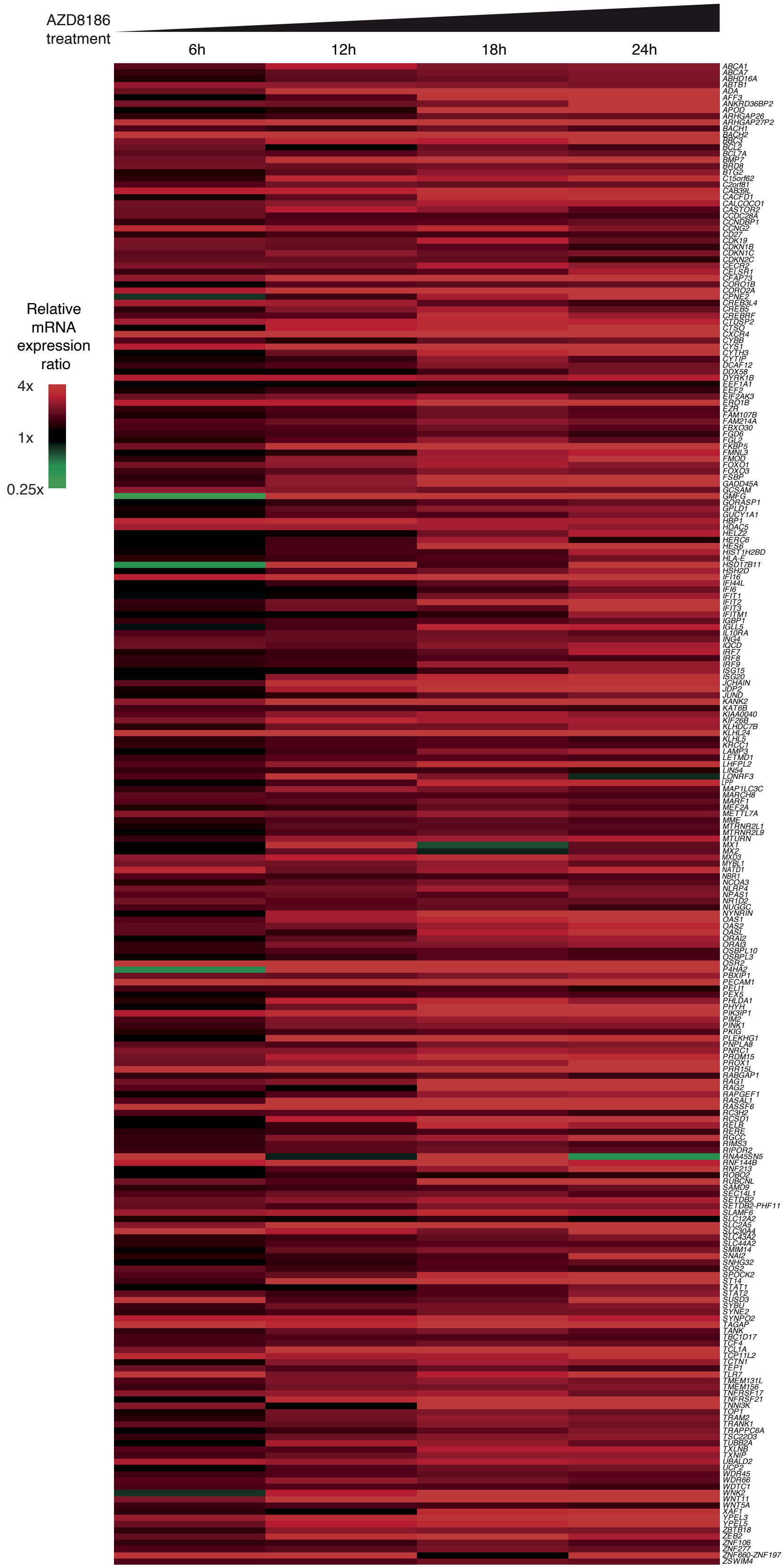

Xu et al. Supplementary Fig. 5

a

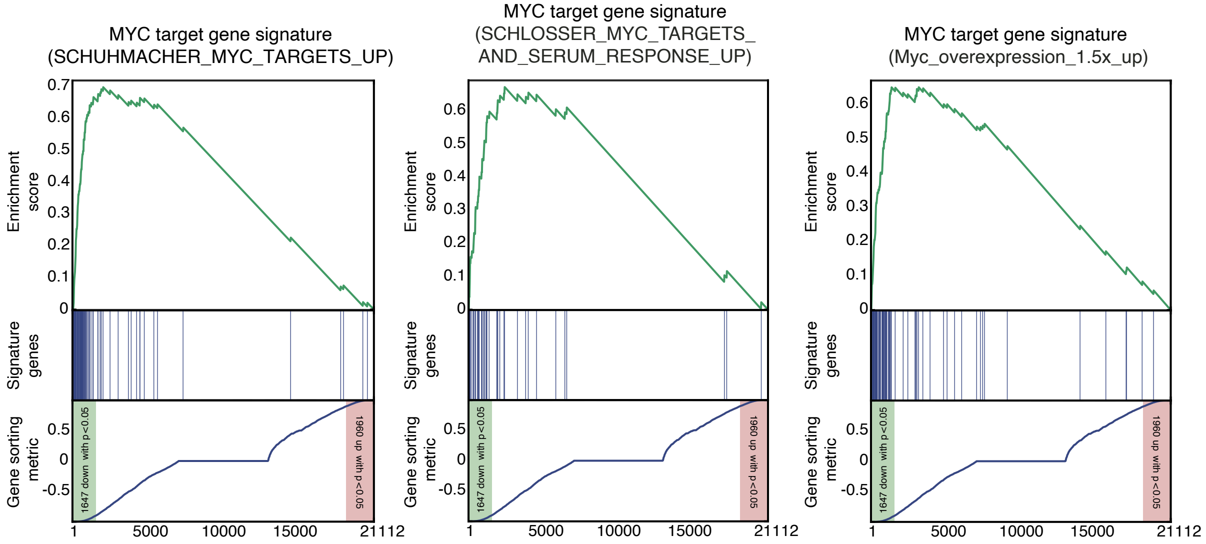

b

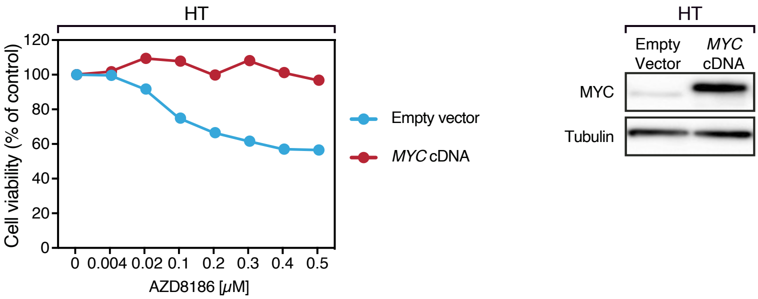

Xu et al. Supplementary Fig. 6

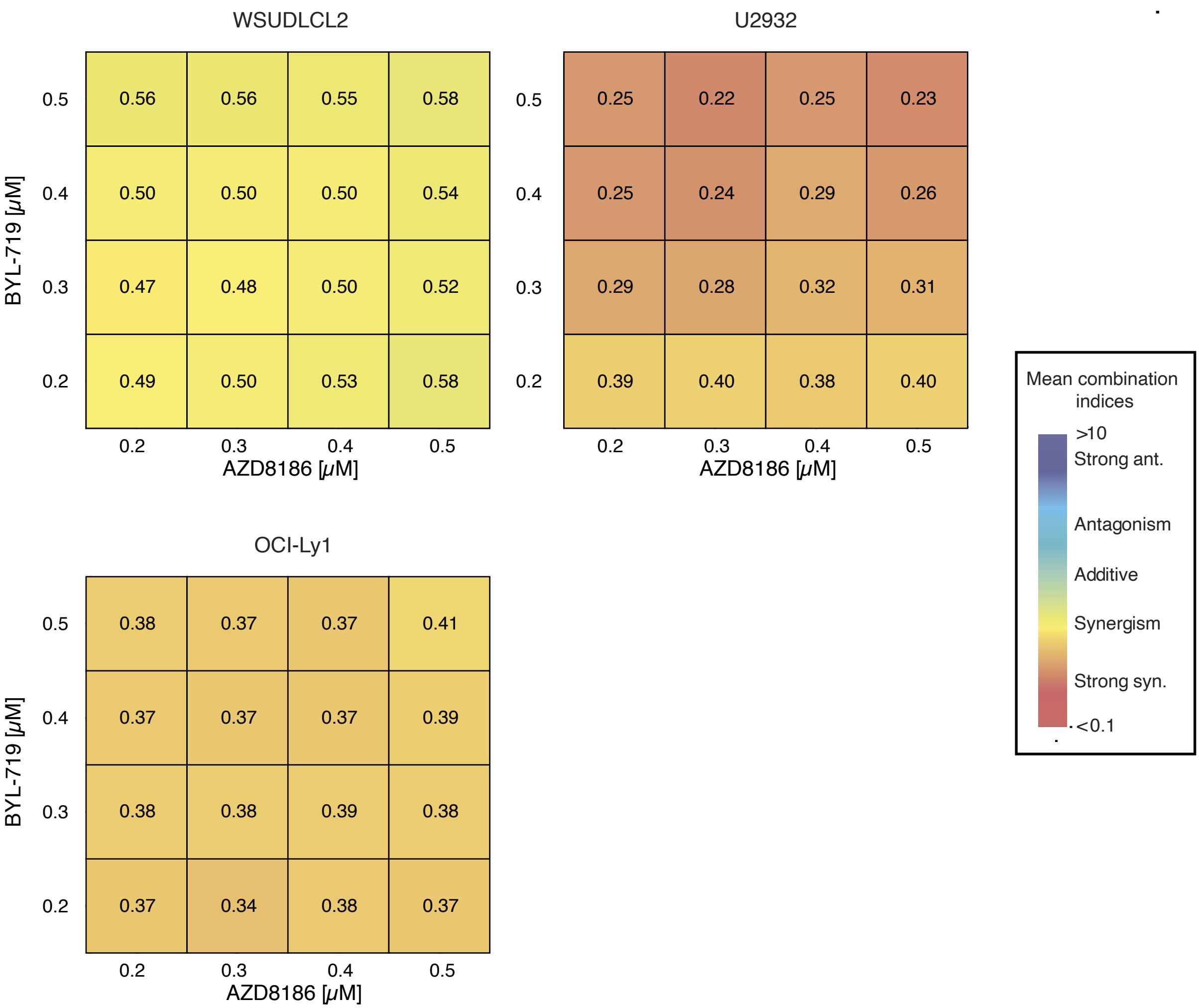

Xu et al. Supplementary Fig. 7

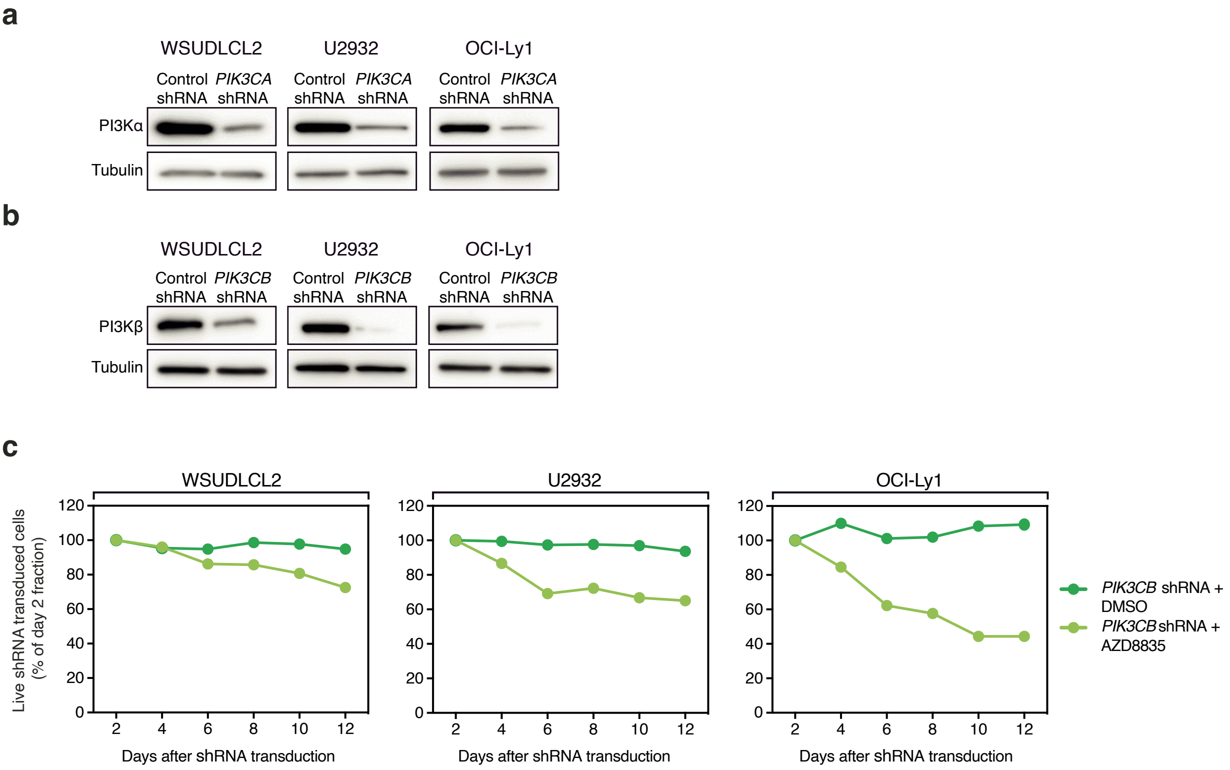

Xu et al. Supplementary Fig. 8

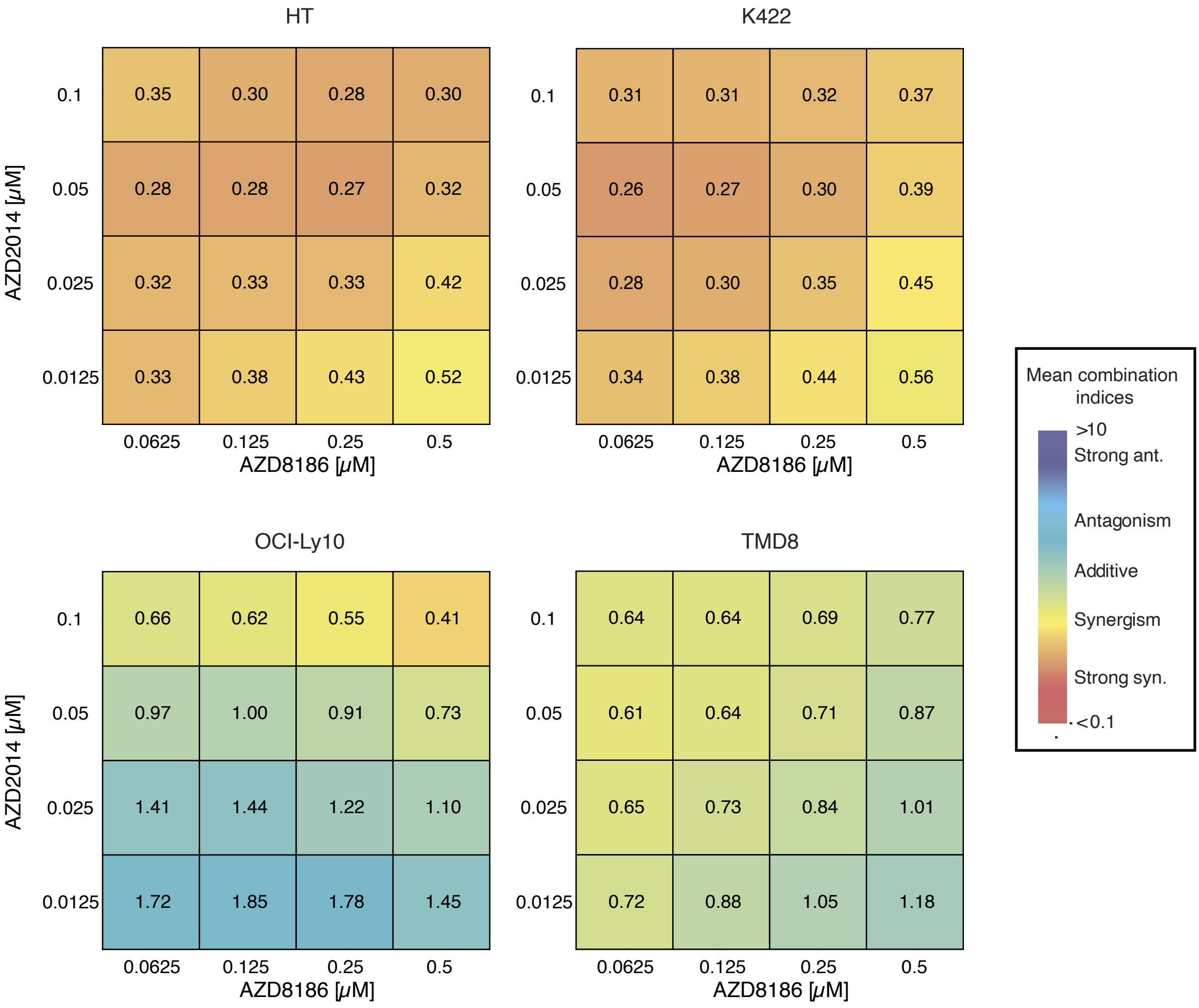

Xu et al. Supplementary Fig. 9

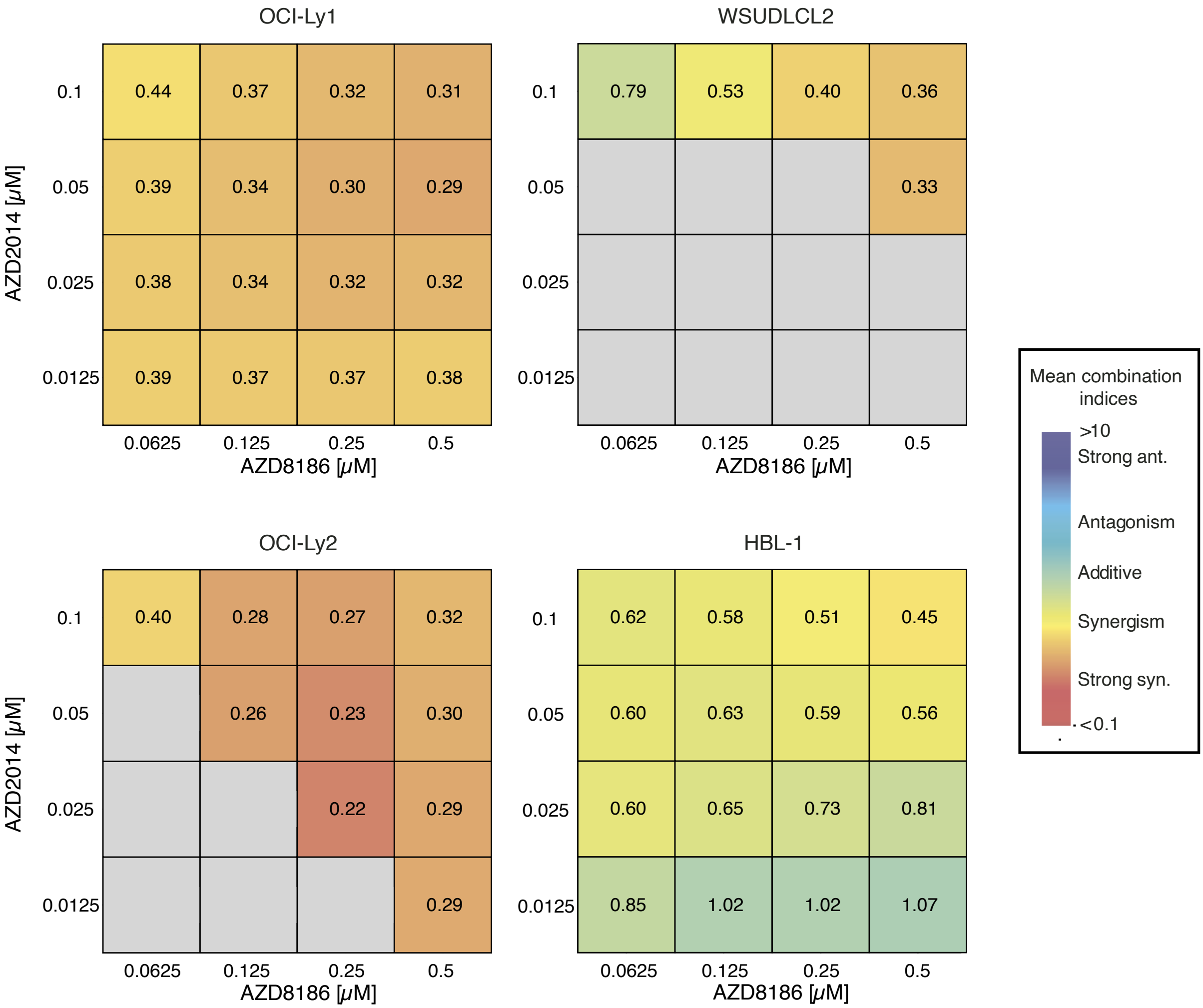

Xu et al. Supplementary Fig. 10

a

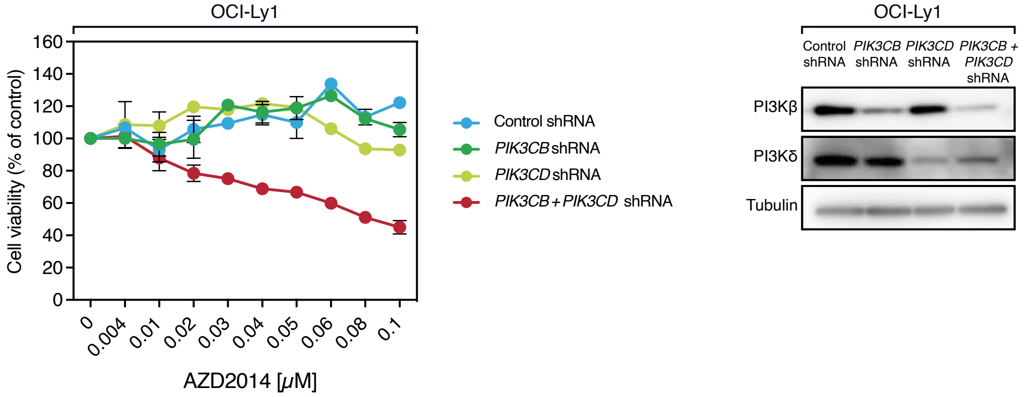

b

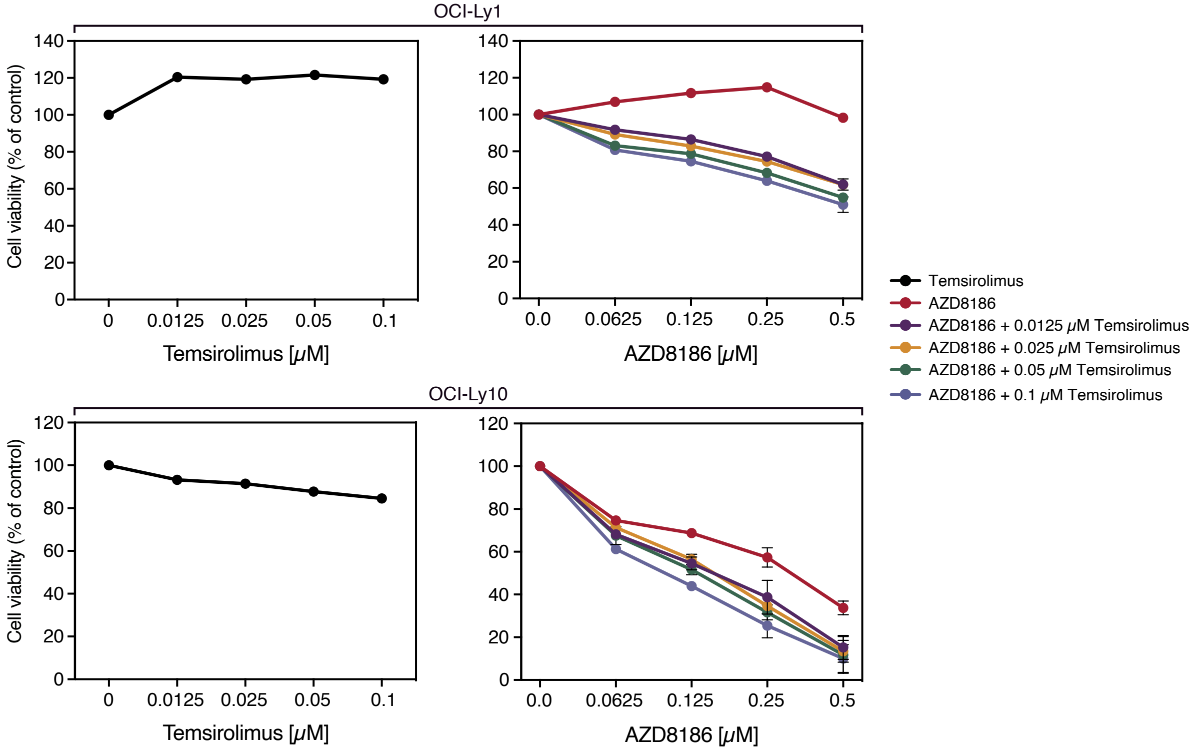

Xu et al. Supplementary Fig. 11

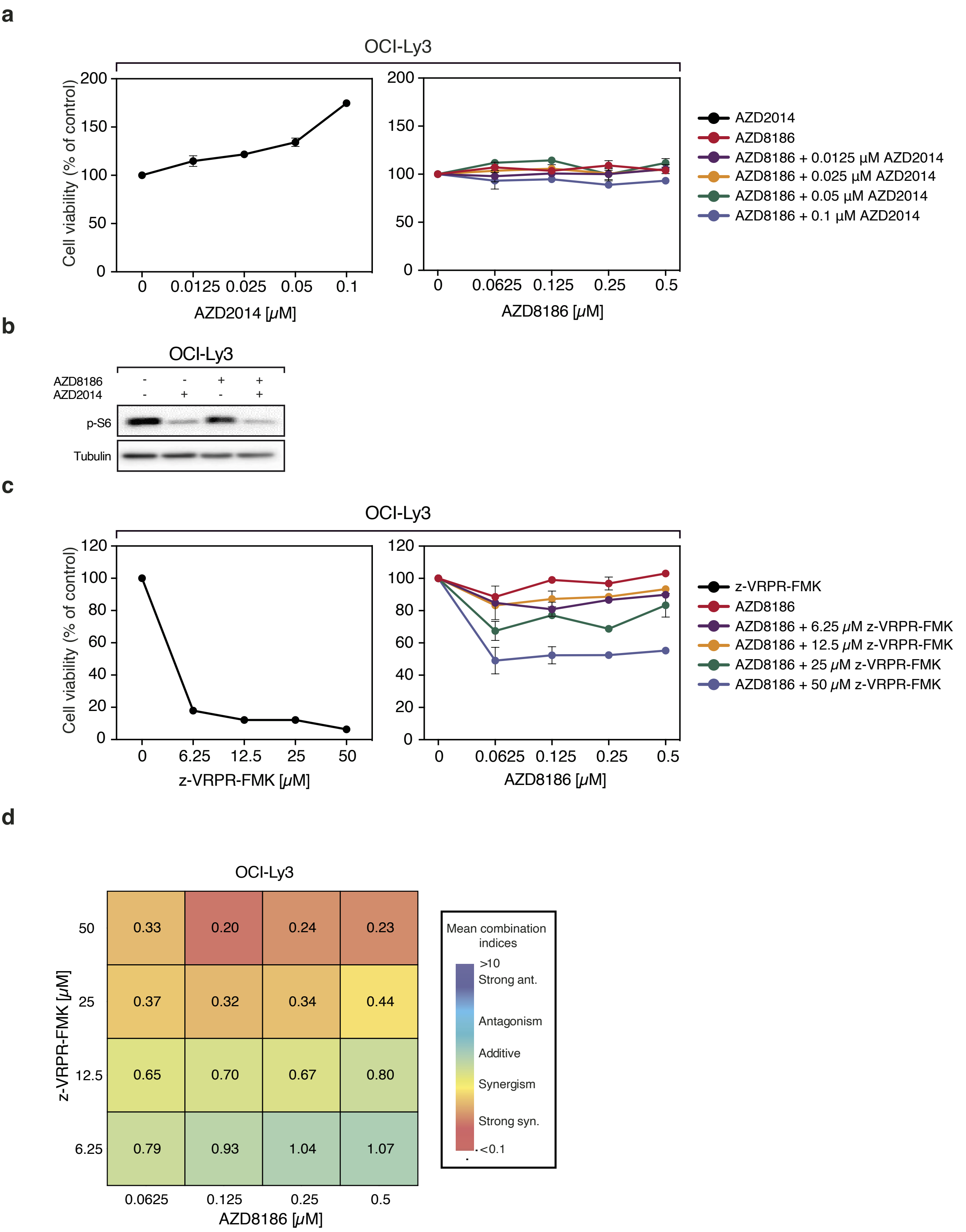

Xu et al. Supplementary Fig. 12

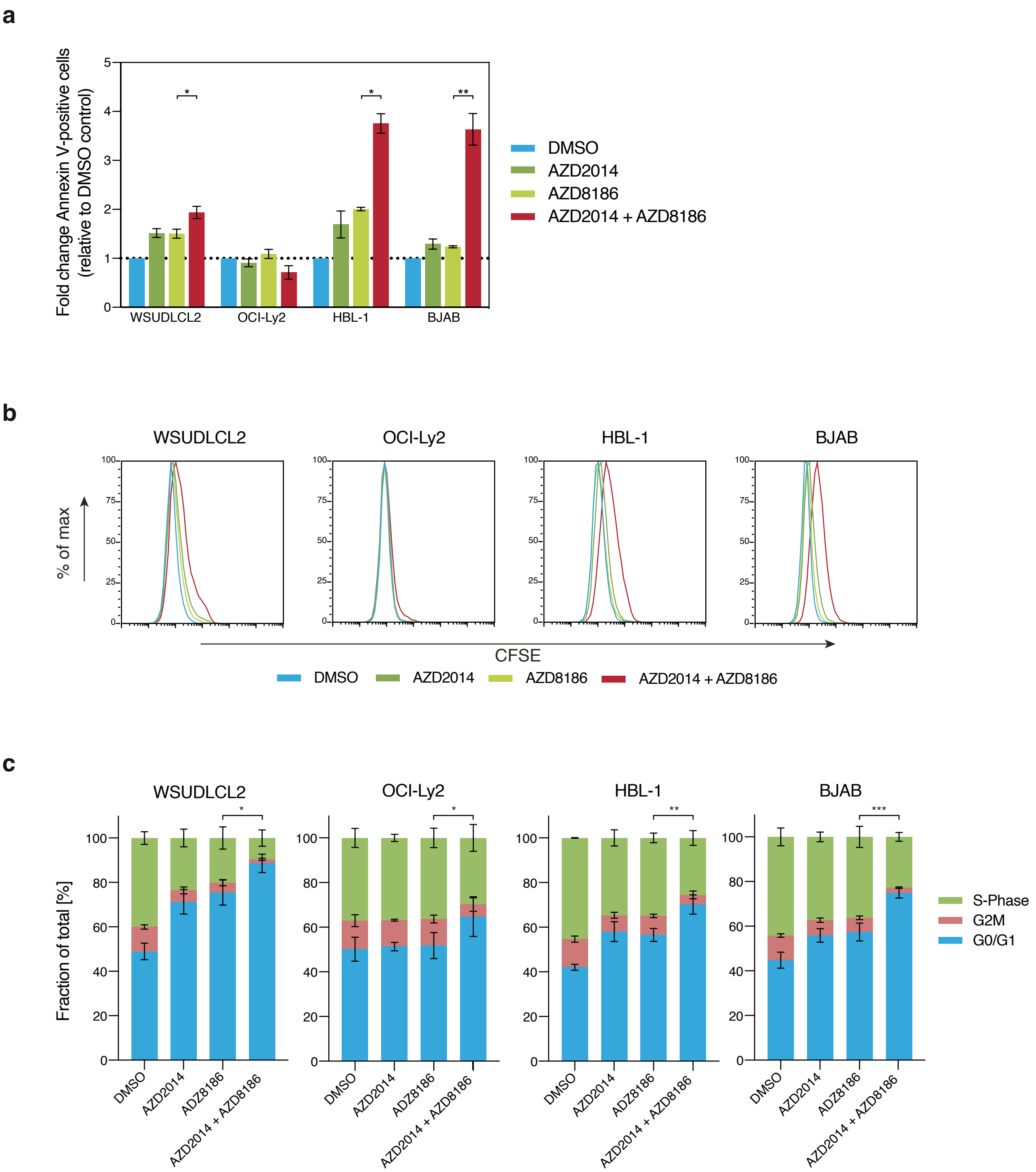

Xu et al. Supplementary Fig. 13

a

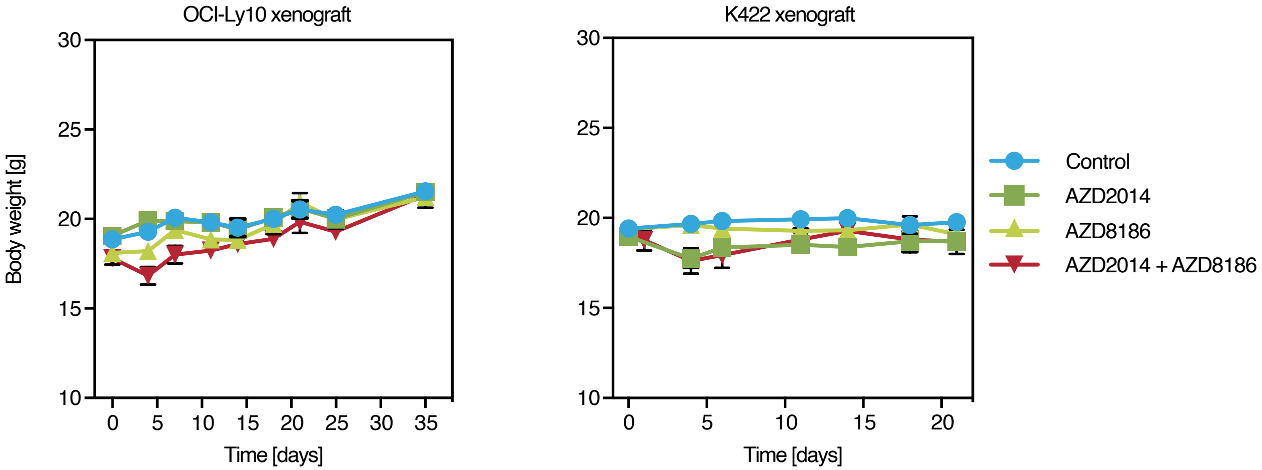

b

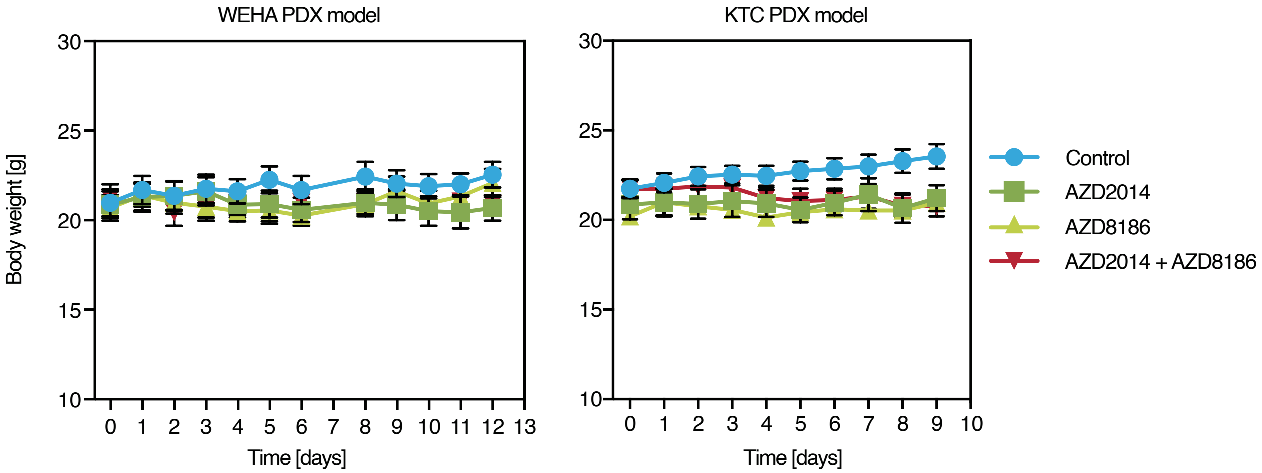

Supplementary Fig. 1. The PI3Kδ inhibitor idelalisib and the PI3Kα/δ inhibitor AZD8835 are effective in subsets of DLBCL.

a-b Cell viability of the indicated DLBCL cell lines was quantified upon single treatment with the indicated concentrations of idelalisib (**a**) or AZD8835 (**b**) after 120 hours. Cell numbers were normalized to the solvent control. Error bars represent standard error of the mean.

Supplementary Fig. 2. Gene expression profiling following AZD8186 treatment in ABC DLBCL.

Heatmap of upregulated genes in OCI-Ly10 and TMD8 cells treated with 0.5 µM of AZD8186 at the indicated time points.

Supplementary Fig. 3. AZD8186 treatment downregulates NF-κB and AP1 signaling in ABC DLBCL.

a A Gene set enrichment analysis revealed multiple NF-κB gene sets downregulated by AZD8186 treatment in OCI-Ly10 and TMD8 cells. **b** Phosphorylation of c-Jun was downregulated faster than its total protein levels in OCI-Ly10 and TMD8 but not in OCI-Ly3 cells after treatment with the pan-PI3K inhibitors GDC-0941 or LY294002. Western blot analyses of phosphorylated c-Jun, c-Jun and phosphorylated AKT (Ser473) after inhibitor treatment for indicated time points are shown. **c** Decrease of c-Jun after AZD8186 treatment is due to proteasomal degradation. Expression levels of c-Jun and phosphorylated AKT (Ser473) were determined by Western blotting in

TMD8 cells after AZD8186 treatment for a total of 4 hours, with or without the proteasome inhibitor MG132 for 2 hours prior to protein harvest.

Supplementary Fig. 4. Gene expression profiling following AZD8186 treatment in GCB DLBCL.

Heatmap of upregulated genes in HT and K422 cells treated with 0.5 μM of AZD8186 at the indicated time points.

Supplementary Fig. 5. AZD8186 treatment downregulates MYC target gene signatures in GCB DLBCL.

a Gene set enrichment analysis revealed multiple previously identified MYC target gene signatures to be downregulated by AZD8186 treatment in HT and K422 cells. **b** Exogenous expression of a *MYC* cDNA completely rescues the AZD8186-sensitive HT cells from AZD8186-induced cytotoxicity. The MYC overexpression was confirmed by western blotting.

Supplementary Fig. 6. AZD8186 and the PI3Kα inhibitor BYL-719 act synergistically in AZD8186-insensitive models.

Mean combination indices are shown as color-coded heatmaps as indicated by the legend. Combination indices were calculated for AZD8186-insensitive cell lines (OCI-Ly1, WSUDLCL2, and U2932) after treatment with AZD8186 and BYL-719 for 120 hours. Data of at least two independent experiments are shown.

Supplementary Fig. 7. PI3K isoforms mediate resistance to PI3Kβ/δ inhibition.

a-b Protein expression of PI3Kα (**a**) and PI3Kβ (**b**) in OCI-Ly1, WSUDLCL2, and U2392 after transduction of a specific shRNA targeting either *PIK3CA* or *PIK3CB*. Western blotting confirm effective knockdown of both isoforms after transduction and doxycycline induction. **c** The indicated DLBCL cell lines were transduced with a vector carrying a *PIK3CB*-specific shRNA which co-expresses green fluorescent protein (GFP). The proportion of GFP-positive cells was quantified after treatment with solvent or 0.8 μM of AZD8835 by flow cytometry and normalized to the control shRNA. For all experiments, the representative data of at least three independent experiments are shown.

Supplementary Fig. 8. AZD8186 and AZD2014 act synergistically in AZD8186-sensitive cell lines.

Mean combination indices are shown as color-coded heatmaps as indicated by the legend. Combination indices were calculated for AZD8186-sensitive cell lines (HT, K422, OCI-Ly10, and TMD8) after treatment with AZD8186 and AZD2014 for 120 hours. Data of at least three independent experiments are shown.

Supplementary Fig. 9. AZD8186 and AZD2014 act synergistically in AZD8186-insensitive cell lines.

Mean combination indices are shown as color-coded heatmaps as indicated by the legend. Combination indices were calculated for AZD8186-insensitive cell lines (OCI-

Ly1, WSUDLCL2, OCI-Ly2, and HBL-1) after treatment with AZD8186 and AZD2014 for 120 hours. For WSUDLCL2 and OCI-Ly2 at lower concentrations, combination indices could not be determined (colored in grey). Data of at least two independent experiments are shown.

Supplementary Fig. 10. Simultaneous silencing of PI3Kβ and PI3Kδ is required to promote AZD2014-induced cytotoxicity.

a AZD8186-resistant GCB DLBCL cell line OCI-Ly1 was transduced with an shRNA targeting *PIK3CB* or *PIK3CD*, or transduced with both shRNAs. Only the double silencing of PI3Kβ and PI3Kδ rendered OCI-Ly1 cells sensitive to AZD2014 treatment, whereas single *PIK3CB* or *PIK3CD*, or a control shRNA-expressing OCI-Ly1 cells remained resistant to AZD2014 treatment. Cells were treated for 120 hours with the indicated AZD2014 concentrations and cell viability was determined by CTG assay. Western blotting confirmed effective knockdown of *PIK3CB* and *PIK3CD*. **b** AZD8186-resistant OCI-Ly1 or sensitive OCI-Ly10 DLBCL cell lines were treated for 120 hours with the indicated AZD8186 and temsirolimus (mTORC1 inhibitor) concentrations and cell viability was determined by CTG assay. For each cell line, left panel shows DMSO-normalized temsirolimus treatment, right panel represents temsirolimus-normalized combination treatments with AZD8186.

Supplementary Fig. 11. MALT1 inhibition sensitizes OCI-Ly3 cells to AZD8186 treatment.

a AZD8186-resistant OCI-Ly3 cells were treated for 120 hours with the indicated AZD8186 and AZD2014 concentrations and cell numbers were determined by the

CTG assay. Left panel shows DMSO-normalized AZD2014 treatment, right panel represents AZD2014-normalized combination treatments with AZD8186. The viability of OCI-Ly3 cells after inhibitor treatment did not decrease, hence no combination indices are available. **b** S6 phosphorylation in OCI-Ly3 cells was analyzed after treatment with either AZD8186 (0.5 µM), AZD2014 (0.1 µM) or with the combination of both drugs for 24 hours. **c** AZD8186 and z-VRPR-FMK combinatorial treatment induces synergistic cytotoxicity in OCI-Ly3 cells. Left panel shows DMSO-normalized z-VRPR-FMK treatment, right panel represents z-VRPR-FMK-normalized combination treatments with AZD8186. **d** Mean combination indices are shown as color-coded heatmaps as indicated by the legend. All data are representative of at least three independent experiments. Error bars correspond to the mean ± SD.

Supplementary Fig. 12. The combination of AZD8186 and AZD2014 induces apoptosis, proliferation arrest and cell cycle inhibition.

a AZD8186 and AZD2014 synergistically induce apoptosis when compared to AZD8186 alone in BJAB, HBL-1, WSUDLCL2, but not in OCI-Ly2 cells. Cells were treated for 48 hours, data are shown as mean ± standard error of the mean after normalization of Annexin V-positive cells to DMSO-treated cells. Representative data of at least two independent experiments are shown. **b** Combination of AZD8186 and AZD2014 inhibits cell proliferation in combination-sensitive cell lines (OCI-Ly2, BJAB, HBL-1, WSUDLCL2). CFSE dilutions are measured at 120 hours of treatment, representative data of at least two independent experiments are shown. **c** AZD8186 and AZD2014 combinatorial treatment induces cell cycle arrest in G0/G1 phase after

24 hours in OCI-Ly2, BJAB, HBL-1, and WSUDLCL2 cells. Means ± SD of at least three independent experiments are shown.

Supplementary Fig. 13. Body weight curves of *in vivo* models after AZD8186 and AZD2014 combination treatment.

Body weights of **a** OCI-Ly10, K422 xenograft mouse models and **b** WEHA, KTC PDX mouse models following treatments of vehicle control (blue), AZD2014 15 mg/kg (dark green), AZD8186 50 mg/kg (light green), and AZD2014 + AZD8186 (red). Treatment was initiated after animals developed macroscopic signs of tumors (day 11 for KTC, day 12 for WEHA after engraftment). Data are shown at indicated timepoints as means ± standard error of the mean.

Supplementary Table 1. Sequences of utilized shRNAs.

Target gene	5’-3’ shRNA sequence
PIK3CA	GGACAACTGTTTCATATAGAT
PIK3CB	GCAACAGCTTTGCATGTTAAA
PIK3CD	GGGTCTTGGGTACGAGAATTC
MSMO1	CTCTCAACCCTTTAAATCTGA

Supplementary Table 2. Western blot antibodies.

Antibody	Source	Provider	Catalogue number
p-AKT (Thr308)	Rabbit monoclonal	Cell Signaling	#2965
p-AKT (Ser473)	Rabbit monoclonal	Cell Signaling	#4058
AKT	Rabbit polyclonal	Cell Signaling	#9272
α-Tubulin	Mouse monoclonal	Sigma	T9026
BCLXL (BCL2L1)	Rabbit monoclonal	Cell Signaling	#2764
p-c-Jun (Ser63)	Rabbit monoclonal	Cell Signaling	#91952
c-Jun	Rabbit monoclonal	Cell Signaling	#9165
c-MYC	Rabbit monoclonal	Abcam	ab32072
IRF4	Rabbit polyclonal	Cell Signaling	#4964
JunB	Rabbit monoclonal	Cell Signaling	#3753
JunD	Rabbit monoclonal	Cell Signaling	#5000
NF-κB p65/RelA	Rabbit monoclonal	Cell Signaling	#8242
NF-κB1 p105/p50	Rabbit polyclonal	Cell Signaling	#3035
PARP	Rabbit polyclonal	Cell Signaling	#9542
PI3 Kinase p110α	Rabbit monoclonal	Cell Signaling	#4249
PI3 Kinase p110β	Rabbit monoclonal	Cell Signaling	#3011
PI3 Kinase p110δ	Rabbit monoclonal	Millipore	#04-401
p-PRAS40 (Thr246)	Rabbit polyclonal	Thermo	44-1100G
PTEN	Mouse monoclonal	Santa Cruz	sc-7974
p-S6 (Ser235/236)	Rabbit polyclonal	Cell Signaling	#2211
